# Postpandemic Use of Video-Based Psychotherapy Among German Outpatient Psychotherapists: Repeated Cross-Sectional and Partially Longitudinal Survey Study

**DOI:** 10.2196/82972

**Published:** 2026-07-31

**Authors:** Julia Rosenbaum, Thomas Berger, Jana Schneider, Michael Spaeth

**Affiliations:** 1 MEU Study Center, DIPLOMA University of Applied Science, Klausenerstrasse 12, Magdeburg, 39112, Germany, 49 159 01237512; 2 Department of Psychology, University of Bern, Bern, Bern, Switzerland; 3 Empowerment Research Institute, Magdeburg, Germany

**Keywords:** video-based psychotherapy, telehealth, teletherapy, digital mental health, technology acceptance, UTAUT, psychotherapist perspective, implementation science

## Abstract

**Background:**

Video-based psychotherapy (VBT) became an essential modality during the COVID-19 pandemic, enabling outpatient care despite social distancing measures. Yet little is known about how usage continued and acceptance evolved after the pandemic. Most existing research is cross-sectional, pandemic-focused, and rarely integrates technology acceptance with clinical process quality and therapist heterogeneity to explain sustained postpandemic VBT use.

**Objective:**

This study examined the postpandemic sustainability of VBT among German outpatient psychotherapists. Guided by UTAUT-T (unified theory of acceptance and use of technology for therapists), we investigated VBT use, acceptance-related predictors, perceived clinical process quality, and therapist acceptance profiles using cross-sectional and longitudinal perspectives.

**Methods:**

We conducted a repeated cross-sectional, partially longitudinal postal survey among licensed German outpatient psychotherapists during the COVID-19 pandemic (T1: July 2020-January 2021) and postpandemically (T2: March-May 2024). The final T2 sample included 296 psychotherapists; 117 participated in both waves. VBT sustainability was assessed through usage status and intensity. Technology acceptance was measured using the UTAUT-T, and clinical process evaluations were assessed using items based on Grawe’s general change mechanisms. Analyses included regression models, longitudinal within-person tests, group comparisons, and person-centered cluster analysis.

**Results:**

Postpandemic VBT use was reported by 68.2% (202/296) of psychotherapists. In retrospective comparisons, use remained above prepandemic levels but below the pandemic peak (4.4% [13/296] prepandemic; 81.1% [240/296] during the pandemic; *χ*^2^_2_=357.2, *P*<.001, Kendall *W*=.64). In the longitudinal subsample, weekly VBT sessions declined from 5.81 during the first pandemic wave to 1.51 postpandemically (*χ*^2^_2_=47.9, *P*<.001, Kendall *W*=.41). UTAUT-T constructs explained 66.6% of the variance in behavioral intention (R²=.666), with therapy quality expectation as the strongest predictor (β=.513, *P*<.001). Behavioral intention predicted postpandemic VBT use (odds ratio 6.56, 95% CI 4.31‐9.99; *P*<.001) and usage intensity (β=.414, *P*<.001). Prior pandemic VBT use and regulatory awareness predicted postpandemic use beyond behavioral intention. Therapists rated VBT as less effective than face-to-face therapy (*z*=13.14, *P*<.001, *r*=0.77), and only 35.8% (106/294) perceived core therapeutic change mechanisms as equally supported. Cluster analysis based on UTAUT-T identified 3 clusters, associated with postpandemic VBT use (*χ*^2^_2_=131.1, *P*<.001, *V*=.67) and significantly differing in pandemic VBT use, age, therapeutic approach, regulatory awareness and restrictions, and perceived equivalence of Grawe’s therapeutic change mechanisms.

**Conclusions:**

This study extends previous pandemic-era and cross-sectional VBT research by examining sustained postpandemic use under more voluntary routine-care conditions and by integrating technology acceptance, clinical process quality, and therapist heterogeneity. The findings show that VBT has become a sustained but selectively used component of German outpatient psychotherapy rather than a universal replacement for face-to-face treatment. Postpandemic use is shaped by perceived clinical meaningfulness, prior experience, regulatory awareness, and therapist acceptance profiles. Implementation efforts should move beyond technical access and support clinically differentiated, profile-sensitive VBT use through targeted training, clear regulatory guidance, and shared decision-making with patients.

## Introduction

The COVID-19 pandemic marked a “black swan” moment for mental health care [1] and accelerated the use of video-based psychotherapy (VBT) in outpatient psychotherapeutic care during physical distancing measures [2-4]. Internationally, VBT use increased substantially during the COVID-19 pandemic [5]. In Germany, more than 40% of sessions were conducted via video during the first lockdown, rising to 57% during the second [6,7]. However, this rapid expansion raises a central implementation question: under what conditions does VBT persist once the extraordinary circumstances that initially facilitated adoption have faded [8,9]? Since the end of pandemic-related restrictions, VBT use has declined, yet not uniformly [10-12], highlighting sustainability as a distinct and dynamic phase of implementation.

Sustainability remains inconsistently defined, ranging from continued delivery to routinization, sustained benefits, adaptation, and contextual alignment [13-17]. In the present study, VBT sustainability is conceptualized as comprising both continued VBT use (breadth) and integration into routine practice (depth), consistent with implementation science frameworks [17-20]. Because binary use and usage intensity may capture different sustainment trajectories, both were assessed [19,21]. In line with Proctor et al [8], adoption refers to initial VBT uptake, sustained use to postpandemic continuation, and implementation to the broader process of integrating VBT into practice.

To understand sustained use, this study is primarily based on the adaptation of the UTAUT-T (unified theory of acceptance and use of technology for therapists) [22,23]. UTAUT-T includes *ease of use*, *therapy quality expectancy*, *professional support*, *pressure from others*, and *convenience* as predictors of technology acceptance [22,23]. In psychotherapy, therapy quality expectancy has consistently emerged as a central predictor of therapists’ intention to use VBT [22,23], suggesting that sustained use depends less on technical feasibility alone than on whether therapists perceive VBT as clinically meaningful, therapeutically effective, and compatible with psychotherapeutic work. This question is especially relevant postpandemically, as most previous studies were conducted during the COVID-19 pandemic, when VBT use was shaped by external constraints and necessity rather than full voluntariness, an important UTAUT moderator [23]. It remains unclear whether pandemic-era acceptance patterns generalize to routine care after restrictions have been lifted [22,24,25]. The German context is particularly relevant because VBT use is embedded in a regulatory framework shaped by certification requirements, quota limitations, and reimbursement structures [26].

Clinical effectiveness is an important but insufficient condition for sustained implementation. Numerous studies indicate that VBT can achieve treatment outcomes broadly comparable to face-to-face (F2F) psychotherapy, particularly for depression and anxiety disorders [27-31]. However, psychotherapists’ acceptance may also depend on whether VBT supports the therapeutic processes they consider central to their work [10,12,32-35]. To examine this clinical dimension, the present study draws on Grawe`s model of general change mechanisms [24,25], which is influential in German-speaking psychotherapy research and practice and provides a transtheoretical framework suitable for a heterogeneous sample with different therapeutic approaches, even if these may differ in the extent to which they emphasize each mechanism [24,25].

Grawe’s model describes 5 general mechanisms of therapeutic change: therapeutic alliance (the quality of collaborative trusting relationship between therapist and patient), problem activation (the deliberate activation of problem-relevant experiences, emotions, and behavioral patterns to facilitate processing and change), motivational clarification (the exploration of clients’ underlying motives, needs, values, and goals), resource activation (the mobilization of clients’ strengths and coping capacities), and mastery (the practice and reinforcement of new behaviors and coping strategies) [24,25].

Emerging evidence suggests differential VBT effects across these mechanisms. Concerns most frequently center on mechanisms relying on physical copresence: problem activation and motivational clarification may be challenged by reduced nonverbal communication and greater relational distance, though VBT may support exposure-based work by enabling patients to engage with feared situations in their everyday environments [10,35-40]. Mastery may be partly facilitated through homework and between-session exercises, though challenges in demonstrating techniques without physical presence have been noted [32]. Regarding the therapeutic alliance, ratings in VBT are marginally lower than in F2F formats but not clinically significant [30,33], and a recent meta-analysis confirmed a positive though somewhat weaker alliance-outcome association than reported for F2F and other online interventions [31], suggesting additional processes may explain outcome variance in VBT. More broadly, therapists report limitations including restricted nonverbal communication, a diminished sense of presence and containment, and perceived incompatibility with core therapeutic values, which may promote more structured, cognitively oriented therapy styles [12,32,35,41-44]. At the same time, VBT introduces distinct affordances: patients often appear more at ease in familiar surroundings [3,41,45], and improved accessibility benefits patients with mobility, time, or geographical constraints [3,33,45,46]. Resource activation appears particularly well-suited to VBT, as access to clients’ home environments provides concrete examples of strengths and support systems [10,32]. VBT should therefore not be understood simply as a digital translation of F2F therapy but as a distinct modality with specific possibilities and constraints. However, most research focuses narrowly on the therapeutic alliance, and postpandemic evidence on broader change mechanisms under voluntary use conditions remains scarce. The absence of longitudinal data limits understanding whether reported constraints reflect genuine limitations of the medium or insufficient therapist adaptation.

Psychotherapists differ in how they evaluate and integrate VBT. Prior studies indicate heterogeneity in technology acceptance, with professional self-doubt, working alliance perceptions, age, therapeutic orientation, and perceived applicability of therapeutic skills shaping VBT acceptance and use [34,47]. However, few studies have used person-centered approaches to identify therapist profiles based on acceptance-related characteristics. Such approaches are useful in implementation research because they capture context-sensitive patterns of attitudes and behaviors rather than isolated predictors [48,49]. The present study therefore applies cluster analysis to examine whether distinct therapist acceptance profiles can be identified and how they relate to postpandemic VBT use, usage intensity, and clinical process evaluations. Identifying such profiles deepens the theoretical understanding of person-technology fit and provides practical implications for targeted training, supervision, and implementation strategies.

This repeated cross-sectional, partially longitudinal survey study examined the postpandemic sustainability of VBT among licensed outpatient psychotherapists in Germany. The study integrates a technology acceptance perspective based on UTAUT-T, a clinical process perspective based on Grawe’s general change mechanisms, and a person-centered perspective on therapist heterogeneity.

First, from a technology acceptance perspective, we examined whether UTAUT-T constructs predicted therapists’ behavioral intention to use VBT and actual postpandemic VBT use, hypothesizing therapy quality expectation as the strongest predictor [12,22]. Second, to specify the clinical dimension, we examined therapists’ evaluations of VBT effectiveness and its perceived capacity to support core change mechanisms. We hypothesized that therapists would evaluate VBT as less effective than F2F therapy, that the therapeutic relationship would be the most frequently compromised change mechanism, and that continued postpandemic users would be more likely than nonusers to perceive VBT as equivalent in addressing core change mechanisms [10,22,33-35,41,46]. Third, to capture heterogeneity beyond a simple user/nonuser distinction, we applied a person-centered approach to identify therapist profiles based on UTAUT-T dimensions, hypothesizing that more than two profiles would emerge and differ in postpandemic VBT use, usage intensity, clinical process evaluations, and therapist characteristics. Fourth, from a longitudinal perspective, we examined how VBT use, usage intensity, motivations, barriers, and perceived effectiveness changed from the pandemic to the postpandemic period, hypothesizing that usage intensity and pandemic-specific motivations would decline as external pressure subsided, while practice-related motivations would become more relevant [8,10-12,16].

Together, these objectives provide a differentiated understanding of how technology acceptance, perceived clinical quality, prior experience, and therapist heterogeneity shape the sustainability of VBT in routine outpatient care. The study results offer empirical guidance for targeted implementation strategies, including therapist-type-specific training, infrastructure support, and regulatory refinement.

## Methods

### Inclusion and Exclusion

Eligible participants were licensed outpatient psychotherapists providing guideline-based psychotherapy for adults in Germany. Inclusion required a valid professional licensure and current or recent involvement in outpatient psychotherapy practice. Participants were excluded if they did not provide information on licensure status.

### Participant Characteristics

The final sample consisted of 296 outpatient German psychotherapists. Sociodemographic and professional characteristics, including age, gender, therapeutic orientation, and years of professional experience, are presented in the results section. Additional variables relevant to the study context included indicators of digital affinity (eg, private and professional internet use) and VBT usage patterns.

### Sampling Procedures

**Figure 1.**
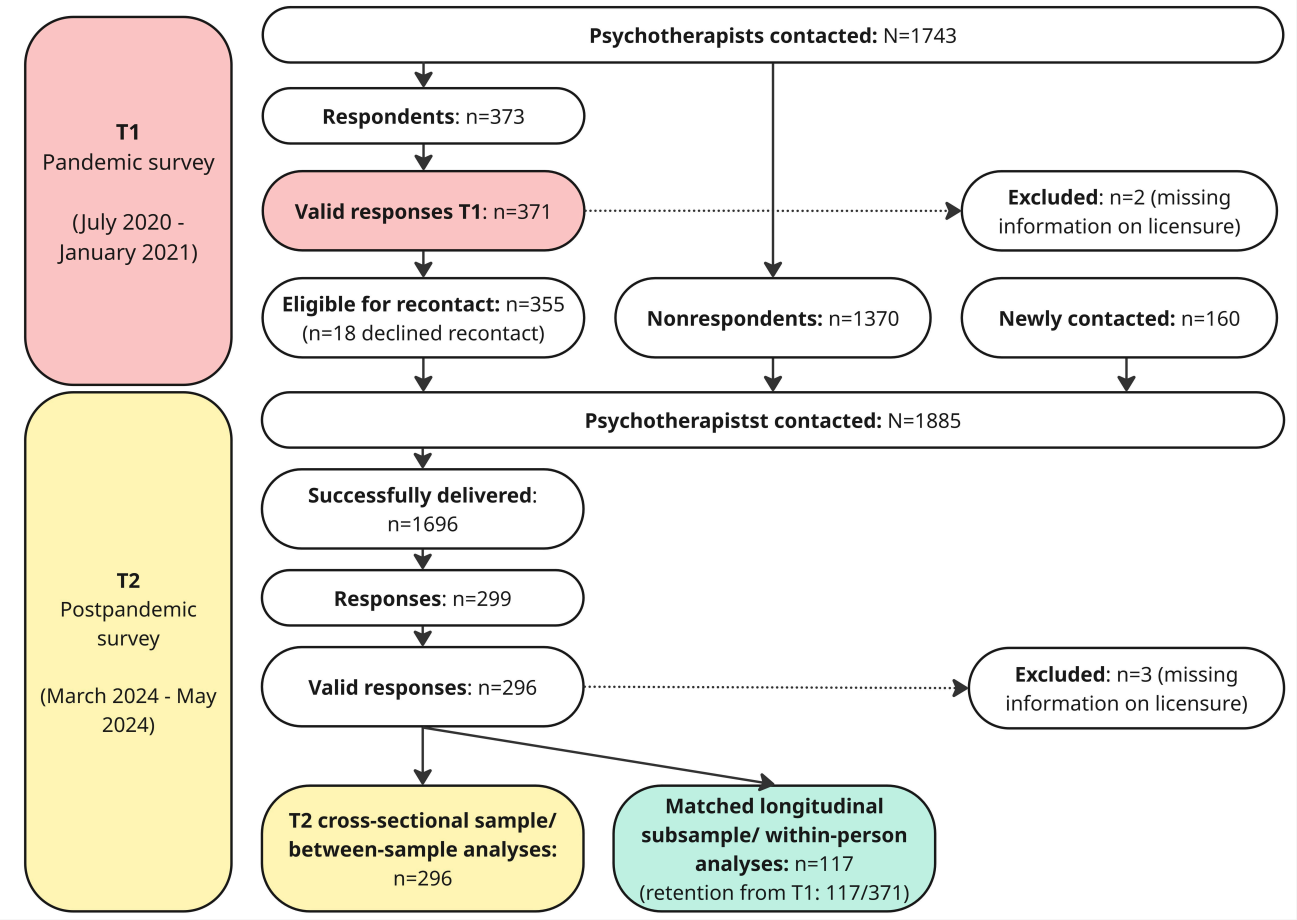
Participant recruitment and analytic samples across survey waves. The figure shows recruitment, sample composition, and analytic samples for the pandemic survey (T1) and postpandemic survey (T2). The T2 sample included a longitudinal subsample for within-person analyses and additional participants for cross-sectional between-sample analyses.

The study was conducted in 5 German federal states organized within the East German Psychotherapists’ Chamber (Ostdeutsche Psychotherapeutenkammer, OPK): Brandenburg, Mecklenburg-Western Pomerania, Saxony, Saxony-Anhalt, and Thuringia. A comprehensive sampling approach aimed at contacting all identifiable licensed outpatient psychotherapists in these regions. Data collection took place between July 2020 and January 2021 for the first survey wave (T1) and between March 2024 and May 2024 for the second survey wave (T2). Recruitment, recontact procedures, and sample composition are shown in Figure 1.

For T2, 1885 psychotherapists were contacted by postal mail, including T1 participants, previous respondents who had not declined recontact, previous nonrespondents, and newly identified practitioners. Of 1696 successfully delivered invitations, 299 questionnaires were returned (response rate: 17.6%), and 296 were retained after excluding 3 responses with missing licensure information. Of these, 117 participants had also participated in T1, forming the matched longitudinal subsample and corresponding to a retention rate of 31.5%.

The response rate is within the expected range for postal surveys of specific professional groups [50,51] and may reflect clinical workload, questionnaire length, and the longitudinal design [52]. The paper-based format was chosen to reduce digital-affinity-related selection bias. Participation was voluntary, resulting in a self-selected sample. T1 data have been reported previously [7]; the present study focuses on postpandemic T2 data and longitudinal changes. Potential attrition bias was assessed by comparing sociodemographic and professional characteristics between longitudinal participants and T1-only participants.

### Ethical Considerations

This study was conducted in accordance with the Declaration of Helsinki and approved by the Ethics Committee of DIPLOMA University of Applied Sciences (reference number: 1104/2024). All participants provided informed consent before participation and were informed about the study’s purpose, procedures, voluntary nature, and their right to withdraw at any time without consequences. Participants from the first survey wave (T1) were informed about possible recontact for follow-up assessments and could explicitly consent to or decline future contact; only those who had not declined recontact were approached for the second survey wave (T2). The informed consent procedure also covered the use of data for longitudinal analyses.

To support recruitment, participants were offered a lottery-based incentive [53,54]. The prize draw included cash prizes of €3000, €2000, and three prizes of €1000 (EUR €1=US $1.0865 as of June 4, 2024), as well as training vouchers worth approximately €200‐250 each for participants ranked 6 to 10. The lottery-based format was chosen as a pragmatic alternative because individual fixed compensation proportionate to outpatient psychotherapists’ opportunity costs was not feasible within the study budget. To ensure transparency, the lottery date was announced in advance, the draw was conducted online, and participants received the link to the online draw together with the study information and questionnaire materials.

Confidentiality and data protection were ensured by collecting identifying information via a separate consent form returned in a different envelope from the survey responses. Survey data were stored separately in pseudonymized form. Longitudinal linkage was enabled through a self-generated pseudonymized identification code based on personal cues, which could not be reconstructed by the research team to identify individual participants [55]. No identifying information is included in this manuscript or supplementary materials, and all results are reported in aggregated form.

### Sample Size, Power, and Precision

We aimed to contact all licensed outpatient psychotherapists within the selected federal states. Thus, no fixed a priori sample size was defined. Instead, the sampling approach approximated a census of the target population within the OPK regions. Given the observational nature of the study, sample size was primarily determined by feasibility and response rates, and no formal a priori sample size calculation was conducted. Sample size adequacy was evaluated based on conventional recommendations for statistical power. For within-subject analyses, a minimum sample size of 44 is required to detect medium effect sizes (*d*=0.5) at α=0.05 with a power of 0.9. For multiple regression analyses with up to 6 predictors, a minimum sample size of 98 is recommended, while larger samples (n≥150) are preferable for stable estimation of smaller effects. The sample size achieved 296 for cross-sectional analyses, and 117 for longitudinal analyses, was considered sufficient to detect medium effects and to conduct regression and subgroup analyses, including cluster analysis.

### Measures and Covariates

The study assessed VBT use, technology acceptance, perceived effectiveness, change mechanisms, and therapist-related characteristics using validated instruments and study-specific items. An overview of all measures, including operationalization and response formats, is provided in Multimedia Appendix 1.

Reflecting heterogeneous approaches to sustainability measurement in prior research [13], VBT sustainability was operationalized through usage persistence and usage intensity. Usage persistence was defined as postpandemic VBT usage status (user vs nonuser), based on self-reported VBT sessions and treated patients. Following established criteria [11], participants were classified as users if they had conducted at least 5 VBT sessions with at least 3 different patients. Usage intensity was captured using the total number of VBT sessions, the number of patients treated via VBT, weekly VBT session frequency, and the proportion of weekly sessions conducted via VBT.

Technology acceptance was assessed using the UTAUT-T framework [22], including the dimensions therapy quality expectation, ease of use, social influence, professional support, convenience, and behavioral intention. Items were rated on a 5-point Likert scale, reversed items were recoded before analysis, and mean scores were computed for each subscale. Perceived effectiveness of VBT compared to F2F psychotherapy was assessed using a single Likert-scale item. Evaluations of therapeutic processes based on Grawe’s general change mechanisms were assessed using multiple-response formats, with each response option coded as a separate dichotomous variable.

Additional variables included prepandemic and pandemic VBT use, digital affinity, awareness of VBT-related regulations, perceived regulatory restrictions, sociodemographic and professional characteristics, and contextual factors such as reasons for VBT use or nonuse. These variables were used as predictors, group comparison variables, or descriptive contextual indicators.

### Data Collection

Data were collected using a structured, paper-based questionnaire distributed by postal mail and returned in prepaid envelopes. The paper-based format was chosen to ensure accessibility regardless of digital affinity. The questionnaire included closed-ended, open-ended, and filter questions, enabling differentiation between VBT users and nonusers. Data collection for T1 took place between July 2020 and January 2021; T2 was conducted between March 2024 and May 2024.

### Quality of Measurements and Instrumentation

The questionnaire was originally developed for the first survey wave to assess psychotherapists’ experiences, attitudes, and use of VBT. Its development involved an expert panel of 5 practicing psychotherapists, some with prior VBT experience. For T2, items were revised or added to reflect the postpandemic context while retaining as many original items as possible to ensure comparability across time points and enable longitudinal analyses. The revised version was again reviewed by 5 experienced psychotherapists. The questionnaire used at T2 is provided in Multimedia Appendix 2.

As data were collected using a standardized self-report questionnaire, no training of data collectors or interrater reliability procedures were required. Measurement quality was supported by clearly defined items and response formats, expert review by practicing psychotherapists, and filter-questions tailored to participants’ VBT experience to reduce response burden and enhance response accuracy.

The questionnaire combined validated instruments and study-specific items. Study-specific items assessed VBT usage patterns, perceived effectiveness, disorder-specific applicability, and evaluations of therapeutic processes based on Grawe’s general change mechanisms [24,25]. Therapists rated whether VBT addresses these 5 mechanisms to a comparable extent as F2F therapy, reflecting subjective professional equivalence ratings. Filter questions were used to tailor item presentation to VBT users and nonusers.

Technology acceptance was assessed with the therapist version of the UTAUT [22], a 21-item self-report measure covering ease of use, pressure from others, therapy quality expectations, professional support, convenience, and behavioral intention. The instrument was translated into German using a forward translation procedure and reviewed by practicing psychotherapists to ensure conceptual equivalence with the original version.

### Masking

Given the observational, nonexperimental design of the study, no masking procedures were used. Participants completed a self-report questionnaire and were fully aware of the study content. No experimental conditions or group assignments were implemented.

### Psychometrics

The UTAUT-T instrument [22] has demonstrated good psychometric properties in previous research, with reported internal consistency coefficients of Cronbach α=0.79 [32]. In the present sample (T2), internal consistency coefficients for the UTAUT-T subscales ranged from α=0.489 to α=0.973, with lower internal consistency observed for some subscales (see Table S1 in Multimedia Appendix 3). Items were aggregated to form scores for each dimension.

Study-specific items assessed perceived effectiveness of VBT, disorder-specific applicability, and evaluations of therapeutic processes based on Grawe’s general change mechanisms [24,25]. Given the exploratory nature of these measures, no composite reliability indices were calculated.

### Conditions and Design

The study used a nonexperimental, observational survey design based on participants’ self-reported experiences and behaviors. It combined repeated cross-sectional and longitudinal elements. Psychotherapists were surveyed at two assessment waves (T1 and T2), allowing comparisons between survey waves. In addition, a matched subsample of participants completed both waves (n=117), enabling within-person analyses of change over time. Accordingly, analyses were conducted at two levels: cross-sectional analyses based on the full T2 sample and longitudinal analyses based on the matched subsample. Throughout the manuscript, T1 and T2 refer to assessment waves, whereas prepandemic, first pandemic phase, second pandemic phase, and postpandemic refer to time-related reference periods assessed within the survey. Comparisons across reference periods are described as longitudinal only when based on the matched subsample with data from both waves. Comparisons based on retrospective reports or nonidentical samples are interpreted as retrospective or repeated cross-sectional comparisons, respectively.

This study was reported in accordance with the STROBE (Strengthening the Reporting of Observational Studies in Epidemiology) guidelines [56]. The completed checklist is provided as Checklist 1.

### Analytic Strategy

Before analysis, participants were excluded if eligibility for the target population could not be verified, particularly if information on licensure status was missing. The missing data assessment therefore refers to the eligible analytic sample. Complete-case analyses with listwise deletion were used as the primary analytic approach. Missing data were low across the analytic variables, ranging from 0% to 1.7% for sociodemographic variables, from 2.4% to 5.1% for items assessing usage frequency, and from 0% to 3% for UTAUT-T items. Little’s test of missing completely at random (MCAR) did not provide evidence against the assumption that data were MCAR (*χ*^2^_175_=199.9; *P*=.10). Multiple imputation with 20 datasets was conducted as a sensitivity analysis for the main regression models using fully conditional specification and predictive mean matching for metric variables. The imputation models included all variables from the respective regressions, and pooled estimates were compared with complete-case results.

Data were screened for outliers using boxplots. No systematic or implausible extreme values requiring exclusion were identified. Descriptive analyses were conducted and nonparametric methods were applied where appropriate. For cluster analysis, variables were *z*-standardized.

The analytic strategy followed the study objectives and distinguished between cross-sectional, longitudinal, retrospective, and exploratory analyses. Cross-sectional T2 analyses examined postpandemic VBT use, group differences between users and nonusers, and determinants of technology acceptance. Group differences were analyzed using chi-square tests and Mann-Whitney *U* tests. Multiple linear regression examined UTAUT-T predictors of behavioral intention, and logistic regression analyses identified factors associated with postpandemic VBT usage status. Regression diagnostics were examined where applicable.

Secondary analyses examined within-person longitudinal changes in the matched subsample using McNemar tests for dichotomous outcomes and Wilcoxon signed-rank tests for ordinal or nonnormally distributed variables. Retrospective comparisons across prepandemic, pandemic, and postpandemic reference periods were based on self-reported T2 data and analyzed using Cochran *Q* tests followed by Bonferroni-adjusted McNemar tests for pairwise comparisons. The retrospective data were not interpreted as true longitudinal measurements. Contextual variables were analyzed longitudinally, where item formats allowed valid comparisons; items introduced only at T2 were reported descriptively.

Exploratory person-centered analyses used a 2-step cluster analysis combining Ward’s method and *k*-means relocation based on *z*-standardized UTAUT-T variables. Therapy quality expectation, ease of use, pressure from others, professional support, and behavioral intention were included; convenience was excluded because it was not predictive of behavioral intention in preliminary analyses. Cluster validity was examined using average silhouette width, the elbow method based on within-cluster sum of squares, and gap statistics. The final solution was selected based on internal validity indices and theoretical interpretability. Cluster differences were examined using ANOVAs or Welch ANOVAs and chi-square tests, with *η*² for ANOVAs and Cramér *V* for chi-square tests reported as effect sizes.

All analyses were conducted using SPSS 29.0 (IBM Inc) and R [57-69]. Statistical significance was defined as a 2-tailed *P* value of <.05.

## Results

### Overview of Analyses

Results are presented in 3 parts. First, sample characteristics and attrition patterns are described. Second, cross-sectional analyses based on the full T2 sample (n=296) provide a detailed picture of postpandemic VBT use, its determinants, and therapists’ evaluations. Third, longitudinal analyses based on the subsample of therapists who participated in both survey waves (n=117) examine how VBT use, underlying motivations, and perceived effectiveness evolved over time.

### Sample and Attrition

At T2, 296 outpatient psychotherapists participated. Of these, 117 also completed T1 and constituted the longitudinal subsample. Table 1 summarizes the sociodemographic and professional characteristics of the full T2 sample, stratified by postpandemic VBT user status. The sample was predominantly female (228/296, 77%), with a mean age of 50.5 (SD 9.2) years, an average of 15.7 (SD 9.2) years of professional experience and 12.6 (SD 8.0) years working in outpatient psychotherapeutic practice. Most participants reported a cognitive behavioral approach (227/296, 76.7%), followed by psychodynamic (70/296, 23.6%), psychoanalytic (12/296, 4.1%), and systemic (7/296, 2.4%) approaches. [In Germany, outpatient psychotherapists are typically trained and licensed in 1 or more of 4 guideline-based therapeutic approaches: cognitive behavioral therapy, psychodynamic therapy, psychoanalysis, and systemic therapy. Membership in one or more of these orientations is required for licensure and reimbursement within statutory health insurance.] Detailed information is provided in Table 1.

**Table 1.** T2 sample characteristics stratified by postpandemic VBT user status^a^.

Characteristics	All	User	Nonuser
Participants, n	296	202	94
Sex, n (%)			
Female	228 (77.0)	158 (78.2)	70 (74.5)
Male	68 (23.0)	44 (21.8)	24 (25.5)
Age in years, mean (SD)	50.52 (9.16)	49.88 (8.98)	51.89 (9.43)
Years working as a psychotherapist, mean (SD)	15.7 (9.2)	15 (8.5)	17.3 (10.4)
Years of working in outpatient psychotherapeutic practice, mean (SD)	12.6 (8.0)	11.9 (7.7)	14.0 (8.5)
Residence, n (%)			
Missing	1 (0.3)	1 (0.5)	0 (0)
Rural area	54 (18.2)	34 (16.9)	20 (21.3)
Small town	54 (18.2)	32 (15.9)	22 (23.4)
Medium-sized town	71 (24.0)	52 (25.9)	19 (20.2)
City	116 (39.2)	83 (41.3)	33 (35.1)
Federal state in which practicing, n (%)			
Brandenburg	58 (19.6)	47 (23.3)	11 (11.7)
Mecklenburg-Western Pomerania	41 (13.9)	27 (13.4)	14 (14.9)
Saxony	89 (30.1)	61 (30.2)	28 (29.8)
Saxony-Anhalt	61 (20.6)	39 (19.3)	22 (23.4)
Thuringia	47 (15.9)	28 (13.9)	19 (20.2)
Therapeutic approach, n (%)			
Cognitive behavioral	227 (76.7)	163 (80.7)	64 (68.1)
Psychodynamic	70 (23.6)	39 (19.3)	31 (33.0)
Psychoanalytic	12 (4.1)	4 (2.0)	8 (8.5)
Systemic	7 (2.4)	5 (2.5)	2 (2.1)
Workload (% of full-time), n (%)			
100%	123 (41.6)	76 (37.6)	47 (50)
75%	5 (1.7)	5 (2.5)	0 (0)
50%	167 (56.5)	119 (58.9)	47 (50)
Private internet use, n (%)			
Hourly	34 (11.5)	24 (11.9)	10 (10.6)
Several times per day	200 (67.6)	146 (72.3)	54 (57.4)
Daily	58 (19.6)	30 (14.9)	28 (29.8)
Weekly	4 (1.4)	2 (1.0)	2 (2.1)
Monthly	0 (0)	0 (0)	0 (0)
Never	0 (0)	0 (0)	0 (0)
Professional internet use compared to colleagues, n (%)			
Missing	5 (1.7)	2 (1.0)	3 (3.2)
Much more	3 (1.0)	3 (1.5)	0 (0)
More	47 (15.9)	42 (20.8)	5 (5.5)
On average	209 (70.6)	141 (69.8)	70 (74.7)
Less	26 (8.9)	14 (6.9)	12 (13.2)
Much less	6 (2.1)	0 (0)	6 (6.6)

a Postpandemic video-based psychotherapy (VBT) use was defined as having conducted at least 5 VBT sessions with at least 3 different patients since April 7, 2023. The total sample included 296 psychotherapists.

Attrition analyses indicated that retained participants were younger (*t*_368_=2.95, *P*=.003, *d*=0.33), with a mean age of 47.56 (SD 8.59) years compared to 50.63 (SD 9.59) years among dropouts, reported fewer years of professional experience (mean 12.41, SD 8.75 vs mean 14.96, SD 9.86; *U*=12,450, *P*=.02, *r*=0.12), and indicated more positive prior experience with VBT (*χ*^2^_3_=10.2, *P*=.02, *V*=.22). No significant group differences were found for pandemic VBT usage, private internet use, perceived VBT effectiveness, intended future VBT use and therapeutic approaches (all *P*>.05). Professional internet use also did not differ significantly between groups (*χ*^2^_4_=7.5, *P*=.11). However, a significant linear-by-linear association was observed (*χ*^2^_1_=4.5, *P*=.03), indicating that lower levels of professional internet use were associated with a higher likelihood of dropout. Detailed results are provided in Table S2 in Multimedia Appendix 3.

### Cross-Sectional Findings

Cross-sectional analyses examined postpandemic VBT use at T2, including usage patterns, its determinants, therapists’ evaluations of clinical process quality, and person-centered profiles of technology acceptance. All analyses are based on T2 data; comparisons across time periods rely on retrospective self-reports.

#### VBT Usage Patterns

Overall, 68.2% (202/296) of therapists reported continued use of VBT after the COVID-19 pandemic, defined as having conducted at least 5 sessions with at least 3 different patients. However, usage intensity was low, averaging 1.9 (SD 2.5) sessions per week, corresponding to 5.7% (SD 6.4) of the total caseload. This corresponded to an average of 50.7 (SD 57.4) sessions conducted with 10.9 (SD 11.3) patients between April 2023 and April 2024.

As shown in Table 2, retrospective comparisons across time periods indicated substantial differences in the proportion of VBT users, increasing markedly from prepandemic levels (13/296, 4.4%) to the pandemic period (240/296, 81.1%), and declining postpandemic (202/296, 68.2%). This retrospective pattern was statistically significant (*χ*^2^_2_=357.2, *P*<.001, Kendall *W*=.64). Post hoc McNemar tests indicated a significant increase from prepandemic use and a significant decrease from pandemic to postpandemic use, with postpandemic use remaining significantly higher than prepandemic use (all *P*<.001, Bonferroni-corrected). The lower postpandemic proportion was primarily due to discontinuation among former users (n=46), whereas only a few therapists newly adopted VBT (n=4).

**Table 2.** Retrospectively reported VBT^a^ user status across reference periods in the T2 sample (n=296).

	Values, n (%)
Prepandemic (cutoff date March 16, 2020)	
User^b^	13 (4.4)
Nonuser^b^	266 (89.9)
Not working as an outpatient^c^	17 (5.7)
Pandemic period (March 16, 2020, to April 7, 2023)	
User^b^	240 (81.1)
Nonuser^b^	50 (16.9)
Not working as an outpatient^c^	6 (2)
Postpandemic (since April 7, 2023)	
User^b^	202 (68.2)
Nonuser^b^	94 (31.8)

a VBT: video-based psychotherapy.

b User status was defined as having conducted at least 5 VBT sessions with at least 3 different patients.

c This category refers to respondents who were not yet practicing as licensed outpatient psychotherapists during the respective reference period but had entered outpatient practice by T2.

#### Technological Acceptance and Predictors of VBT Use

UTAUT-T constructs showed strong associations with therapists’ intention to use VBT.

Correlation analyses indicated that behavioral intention was most strongly associated with therapy quality expectation (*r*=0.76, *P*<.001, n=296), followed by ease of use (*r*=0.60, *P*<.001, n=296), pressure from others (*r*=0.51, *P*<.001, n=292), and professional support (*r*=0.43, *P*<.001, n=296). Convenience showed a weaker but still significant association with behavioral intention (*r*=0.33, *P*<.001, n=296).

A multiple regression analysis (n=292) confirmed the predictive relevance of UTAUT-T constructs for behavioral intention (*F*_5,286_=113.91, *P*<.001, *R*²=.666, adjusted *R*²=.660). Therapy quality expectation (β=.513, *P*<.001) and ease of use (β=.213, *P*<.001) emerged as the strongest predictors. Pressure from others (β=.197, *P*<.001) and professional support (β=.108, *P*=.005) also contributed significantly, whereas convenience did not (β=−.006, *P*=.87). No multicollinearity issues were observed (tolerance>0.4; variance inflation factor [VIF]<2.5).

To examine if behavioral intention translates into actual use, a binary logistic regression was conducted with postpandemic VBT usage status as the dependent variable. The model was statistically significant (*χ*^2^_1_=176.8, *P*<.001), explained 63% of the variance (Nagelkerke *R*²=.630), and correctly classified 89.2% of cases (n=296). Behavioral intention significantly predicted postpandemic VBT use (odds ratio [OR] 6.56, 95% CI 4.31-9.99; *P*<.001). Behavioral intention significantly predicted the proportion of VBT sessions, *F*_1,232_=47.97, *P*<.001, explaining 17.1% of the variance (*R*²=.171). Higher behavioral intention was associated with a higher proportion of weekly VBT sessions (*B*=2.51, SE=0.36; β=.414, 95% CI 1.80-3.23; *P*<.001). A sensitivity analysis using Spearman rank correlation confirmed this association (ρ=0.565, 95% CI 0.468-0.649; *P*<.001; n=234), indicating that the relationship was robust to deviations from normality.

To explore predictors of postpandemic VBT usage status, a binary logistic regression was conducted with theoretically relevant predictors entered simultaneously (Table 3). The model was statistically significant (*χ*^2^_9_=180.3, *P*<.001), explained 68.7% of the variance (Nagelkerke *R*²=.687), and correctly classified 88.9% of cases. Behavioral intention was the strongest predictor. Prior pandemic VBT use and greater awareness of VBT regulations also remained significant predictors. Age showed only a weak and less robust association. Perceived effectiveness, perceived equivalence of Grawe’s change mechanisms, perceived regulatory restrictions, professional internet use, and therapeutic approach were not significant independent predictors.

Multiple-imputation sensitivity analyses supported the complete-case findings: the same predictors remained significant in the UTAUT-T regression, and behavioral intention, prior pandemic VBT use, and regulatory awareness remained significant predictors of postpandemic VBT use.

**Table 3.** Binary logistic regression predicting postpandemic VBT usage status^a^ (n=270).

Predictor	*B*	SE	Wald chi-square (*df*)	*P* value	OR^b^ (95% CI)
Pandemic VBT use^c^	1.812	0.758	5.71 (1)	.02	6.12 (1.38-27.07)
Behavioral intention	1.661	0.297	31.38 (1)	<.001	5.27 (2.95-9.42)
Awareness of VBT regulations^d^	−0.944	0.348	7.34 (1)	.007	0.39 (0.20-0.77)
Perceived effectiveness of VBT^e^	0.368	0.447	0.68 (1)	.44	1.44 (0.60-3.47)
Grawe’s change mechanisms^e^	0.074	0.514	0.02 (1)	.89	1.08 (0.39-2.95)
Perceived regulatory restrictions^d^	−0.252	0.235	1.15 (1)	.28	0.78 (0.49-1.23)
Professional internet use	−0.127	0.457	0.08 (1)	.78	0.88 (0.36-2.16)
Age	0.059	0.030	3.88 (1)	.049	1.06 (1.00-1.13)
Therapeutic approach CBT/PD^f^	−0.563	0.541	1.08 (1)	.30	0.57 (0.20-1.65)

a Postpandemic video-based psychotherapy (VBT) use was coded as 0=nonuser and 1=user.

b OR: odds ratio.

c Pandemic VBT use was coded as 0=nonuser and 1=user.

d For regulatory awareness and perceived restrictions, higher values indicate lower agreement (1=strongly agree, 4=strongly disagree); therefore, odds ratios (ORs) below 1 indicate that greater awareness was associated with higher odds of postpandemic VBT use.

e For perceived effectiveness, higher values indicate lower perceived effectiveness (1=much better, 5=much worse).

f Therapeutic orientation was coded as 0=cognitive behavioral therapy (CBT) and 1=psychodynamic therapy (PD).

#### Perceived Effectiveness, Clinical Process Quality, and User Experiences

Analyses in this section address the clinical process perspective by examining therapists’ evaluations of VBT effectiveness, perceived support of core therapeutic change mechanisms, and user experiences.

Therapists rated VBT as significantly less effective than F2F therapy, with a large effect (*z*=13.14, *P*<.001, *r*=0.77, n=290). Effectiveness ratings did not differ by therapeutic orientation (U=6391.50, *P*=.14, *r*=0.09) but differed significantly by postpandemic usage status (U=5638.00, *P*<.001, *r*=0.36), with nonusers evaluating VBT less favorably than users.

Evaluations of Grawe’s general change mechanisms showed a similar pattern. Overall, 106/294 (35.8%) therapists indicated that VBT addressed core change mechanisms comparably to F2F therapy, whereas 187/294 (63.2%) reported limitations. The most frequently affected mechanisms were the therapeutic relationship (57.8% [171/296]), problem activation (30.1% [89/296]), and motivational clarification (13.9% [41/296]). Users endorsed equivalence more often than nonusers (91/202, 45% vs 15/94, 16%; *χ*^2^_1_=26.8, *P*<.001, *V*=.30). Nonusers also more often rated the therapeutic relationship as weaker in VBT than in F2F settings (74/76, 97.4% vs 97/110, 88.2%, *χ*^2^_1_=5.1, *P*=.03, *V*=.17).

Despite these reservations, overall user experiences with VBT were predominantly positive. Among users, 138/202 (68.3%) reported positive or very positive experiences, 62/202 (30.7%) reported mixed experiences, and only 2/202 (1%) reported negative experiences (mean 2.19, SD 0.69, 1=very positive, 5=very negative). 135/202 (66.8%) users reported that VBT supported the therapeutic process, and 145/202 (71.8%) observed positive patient responses. However, only 47/202 (23.3%) reported clear therapeutic gains, whereas 40/202 (19.8%) reported no observable effect and 7/202 (3.5%) reported negative outcomes. Although a minority of users (15/202, 7.4%) reported feeling overwhelmed or dissatisfied, negative patient feedback was rare (1/202, 0.5%). Users also reported several challenges conducting VBT. The most frequently reported concerns were the potential reinforcement of avoidance behavior (100/202, 49.5%), reduced perceptual engagement (94/202, 46.5%), and limited eye contact (52/202, 25.7%).

#### Therapist Heterogeneity and Person-Centered Profiles

**Figure 2.**
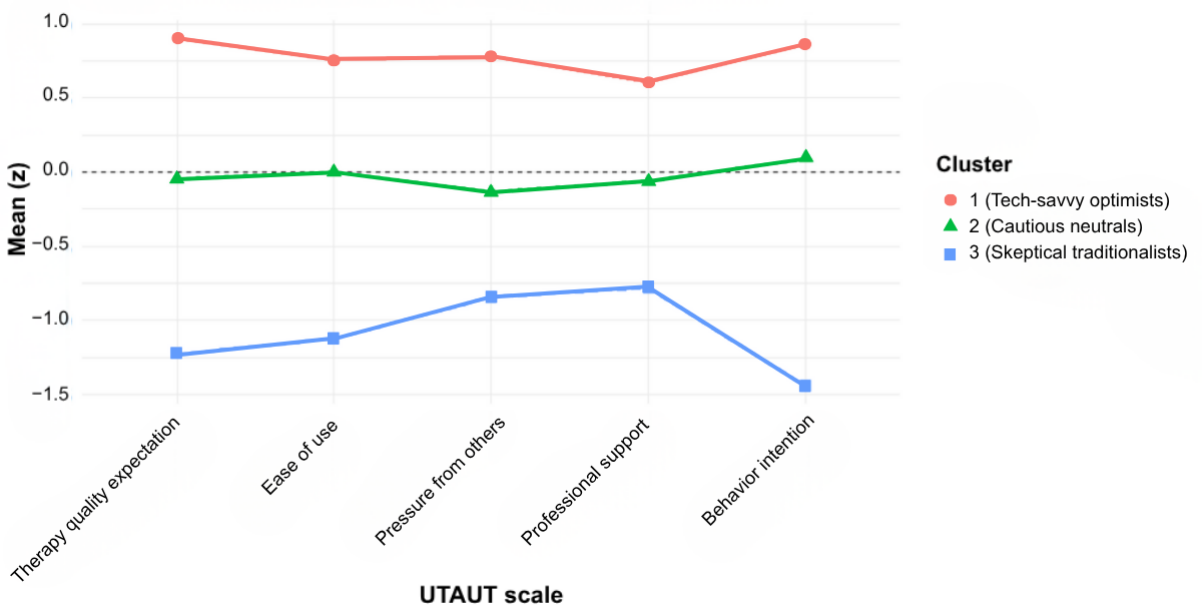
*Z*-standardized cluster profiles across UTAUT-T scales. Higher values indicate higher therapy quality expectation, ease of use, pressure from others, professional support, and behavioral intention. UTAUT-T: unified theory of acceptance and use of technology for therapists.

A 2-step cluster analysis based on 5 predictive UTAUT-T variables (therapy quality expectation, ease of use, pressure from others, professional support, and behavioral intention) identified a 3-cluster solution. The solution showed limited-to-moderate separation (average silhouette width *M*=0.22) but was retained because it provided theoretically interpretable acceptance profiles beyond a simple user/nonuser distinction. Figure 2 illustrates the *z*-standardized cluster profiles.

Cluster 1 (*tech-savvy optimists*; 95/292, 32.5%) was characterized by high perceived usefulness, high ease of use, and strong behavioral intention. Cluster 2 (*cautious neutrals*; 130/292, 44.5%) showed moderate levels across all UTAUT-T dimensions, indicating ambivalent attitudes and moderate intention to use VBT. Cluster 3 (*skeptical traditionalists*; 67/292, 23%) was characterized by low perceived usefulness, low ease of use, and low behavioral intention.

A multivariate analysis of variance confirmed significant differences between clusters across all UTAUT-T dimensions (Pillai’s Trace =0.81, *F*_10,574_=55.14, *P*<.001). Univariate analyses showed significant between-cluster differences for all variables (all *P*<.001), with large effect sizes (*η*² range=.266-.726; Table 4). Post hoc comparisons consistently indicated a gradient pattern (cluster 1>cluster 2>cluster 3), with the largest differences observed for behavioral intention. Cluster membership was strongly associated with postpandemic VBT usage status (*χ*^2^_2_=131.1, *P*<.001; *V*=.67, 95% CI 0.57-1; n=292), indicating a large effect. A clear gradient emerged across clusters, with *tech-savvy optimists* showing the highest user rates, followed by *cautious neutrals*, and substantially lower rates among *skeptical traditionalists* (see Table 5). Post hoc comparisons confirmed significant differences between all cluster pairs (all *P*≤.001).

**Table 4.** UTAUT-T^a^ scale differences across therapist clusters (n=292)^b^.

Variable	*F* (*df*)	*P* value	*η*²
Ease of use	135.0 (2, 289)	<.001	.482
Therapy quality expectation	230.0 (2, 289)	<.001	.614
Pressure from others	85.8 (2, 289)	<.001	.373
Professional support	52.3 (2, 289)	<.001	.266
Behavioral intention	384.0 (2, 289)	<.001	.726

a UTAUT-T: unified theory of acceptance and use of technology for therapists.

b All pairwise between-cluster comparisons were statistically significant after adjustment for multiple testing (adjusted *P*<.001).

**Table 5.** Postpandemic VBT user status by cluster membership (n=292)^a^.

Cluster	Cluster size, n (%)	VBT user rate, n (%)
1. Tech-savvy optimists	95 (32.5)	88 (92.6)
2. Cautious neutrals	130 (44.5)	103 (79.2)
3. Skeptical traditionalists	67 (22.9)	8 (11.9)

a Cluster membership was associated with postpandemic video-based psychotherapy (VBT) user status, *χ*2_2_=131.1, *P*<.001, Cramér *V*=.67 (95% CI 0.57-1).

A similar gradient pattern was observed for VBT usage intensity. Clusters differed significantly across all usage indicators (all *P*<.001), with *tech-savvy optimists* reporting the highest levels of use, followed by *cautious neutrals*. In contrast, *skeptical traditionalists* consistently reported minimal use (see Table 6).

**Table 6.** Postpandemic VBT^a^ usage intensity by cluster membership (n=292).

Cluster	Total sessions, mean (SD)	Total patients, mean (SD)	Weekly sessions, mean (SD)	Weekly caseload (%), mean (SD)
1. Tech-savvy optimists	66.84 (72.91)	13.07 (12.98)	2.24 (2.15)	8.09 (7.75)
2. Cautious neutrals	30.29 (29.55)	7.31 (8.79)	1.42 (2.71)	3.15 (3.46)
3. Skeptical traditionalists	15.00 (22.76)	4.14 (6.31)	0.55 (0.80)	1.73 (3.32)

a VBT: video-based psychotherapy.

This pattern extended to prior experience and demographic characteristics (see Table 7). Pandemic VBT use varied markedly across clusters (*χ*^2^_2_=82.3, *P*<.001, *V*=.53), with nearly all therapists in the two more accepting clusters reporting prior use, compared to less than half of *skeptical traditionalists* (all adjusted *P*≤.006). Age also differed significantly (Welch ANOVA: *F*_2,154.23_=6.09, *P*=.003, *η*²=.05), with *skeptical traditionalists* being older than both *tech-savvy optimists* and *cautious neutrals* (*P*≤.02). The therapeutic approach was associated with cluster membership (*χ*^2^_2_=12.4, *P*=.002, *V*=.19), with the highest proportion of cognitive behavioral therapists among *cautious neutrals* and the lowest among *skeptical traditionalists*; this difference was significant between these two clusters (adjusted *P*=.001). Professional internet use showed a strong association with cluster membership (H(2)=52.1, *P*<.001, *η*²=.17), following an inverse gradient pattern (*skeptical traditionalists*>*cautious neutrals*>*tech-savvy optimists*). Awareness of VBT regulations differed across clusters (H(2)=24.3, *P*<.001, *η*²=.08), with lower awareness among *skeptical traditionalists* than in the other clusters. A comparable pattern was observed for perceived regulatory restrictions (H(2)=32.6, *P*<.001, *η*²=.11), with *skeptical traditionalists* reporting fewer perceived restrictions. Finally, evaluations of clinical processes differed systematically. Perceived equivalence of Grawe’s therapeutic change mechanisms was highest among *tech-savvy optimists* and decreased across clusters, with significant differences between all groups (all adjusted *P*≤.001). No differences were observed for prior VBT training (*χ*^2^_2_=0.5, *P*=.79, *V*=.0).

**Table 7.** Therapist and VBT-related characteristics by cluster membership (n=292)^a^.

Variable	Tech-savvy optimists	Cautious neutrals	Skeptical traditionalists
Age (years), mean (SD)	48.64 (9.86)	50.12 (8.07)	53.91 (9.46)
Therapeutic approach CBT^b^, n (%)	74 (77.7)	110 (84.5)	42 (62.1)
Pandemic usage rate, n (%)	94 (98.9)	117 (89.7)	31 (46.2)
Change mechanisms equally, n (%)	57 (60.0)	41 (31.5)	7 (10.6)
Prior VBT training, n (%)	15 (16.0)	20 (15.0)	8 (12.1)
Awareness of VBT regulations, median (IQR)	4 (1)	4 (1)	3 (1)
Perceived regulatory restrictions, median (IQR)	3 (1)	3 (1)	1 (1)
Professional internet use, median (IQR)	3 (1)	3 (1)	3 (2)

a For median-based variables, higher values indicate higher levels of the respective construct. Awareness of video-based psychotherapy (VBT) regulations and perceived regulatory restrictions were recoded so that higher values indicate stronger agreement. Awareness and perceived restrictions were rated on a 4-point scale; professional internet use was rated on a 5-point scale.

b CBT: cognitive behavioral psychotherapy.

### Longitudinal Changes in VBT Use

Longitudinal analyses extend the cross-sectional findings by examining changes in VBT use, usage intensity, underlying motivations and barriers, and perceived effectiveness among therapists who participated in both survey waves (n=117).

#### VBT Usage Over Time

**Figure 3.**
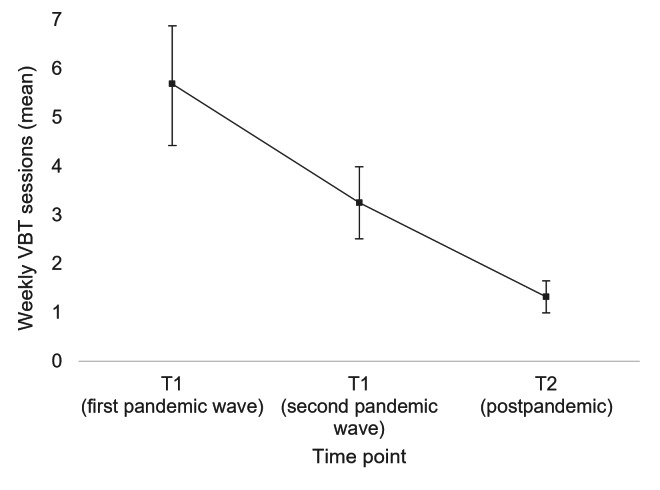
Average weekly video-based psychotherapy (VBT) sessions across time points. Values represent the mean number of VBT sessions per week, with 95% CIs. Values for the first and second pandemic reference periods were reported at T1; postpandemic values were assessed at T2 in the same participants. Thus, the figure combines retrospectively reported pandemic reference-period data with postpandemic follow-up data from the matched longitudinal subsample.

**Figure 4.**
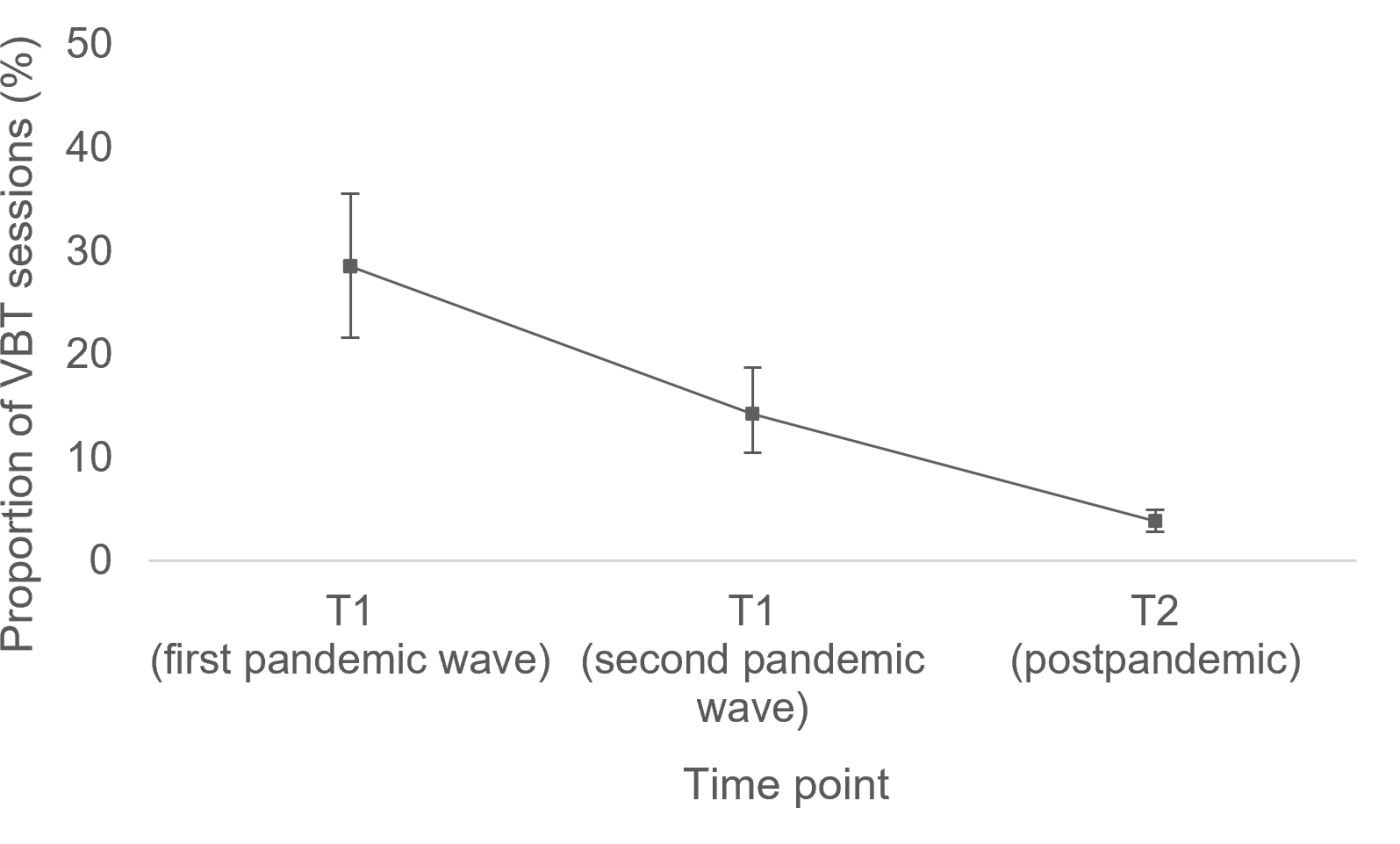
Proportion of video-based psychotherapy (VBT) sessions across time points. Values represent the mean percentage of weekly sessions conducted via VBT, with 95% CIs. Values for the first and second pandemic reference periods were reported at T1; postpandemic values were assessed at T2 in the same participants. Thus, the figure combines retrospectively reported pandemic reference-period data with postpandemic follow-up data from the matched longitudinal subsample.

**Figure 5.**
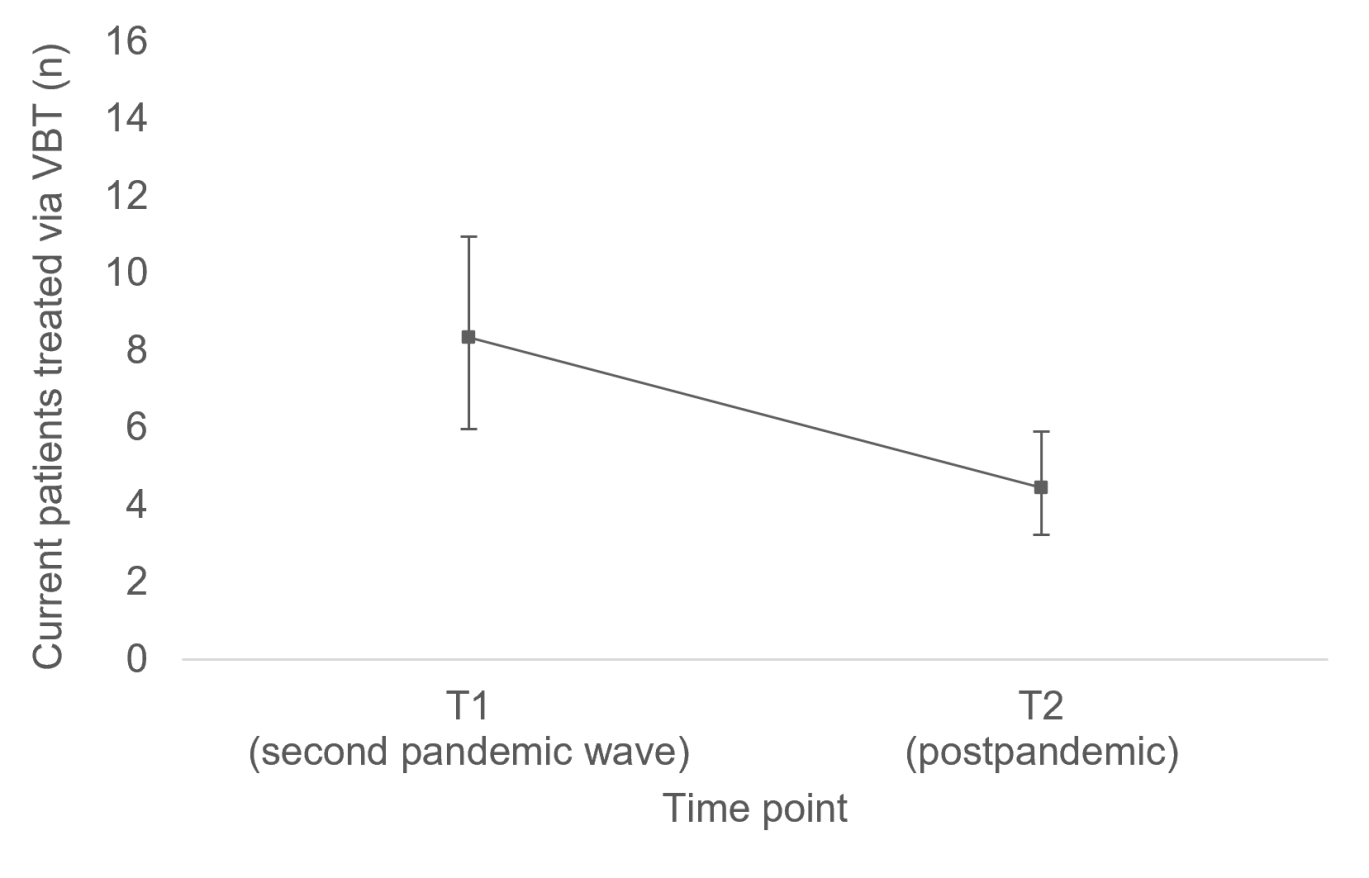
Number of current patients treated via video-based psychotherapy (VBT) across time points. Values represent the mean number of current patients who received at least 1 VBT session, with 95% CIs. Pandemic values were reported at T1, and postpandemic values were assessed at T2 in the matched longitudinal subsample.

The proportion of therapists using VBT remained stable over time. Of the 117 therapists, 15 (12.8%) initiated VBT use postpandemic, whereas 14 (12%) discontinued use, resulting in no significant net change (McNemar test: *χ*^2^_1_=0, *P*>.99). In contrast, usage intensity declined markedly. Weekly VBT sessions decreased significantly across time points (Friedman test: *χ*^2^_2_=47.9, *P*<.001, Kendall *W*=.41, indicating a large effect), from mean 5.81 (SD 5.30) during the first pandemic wave to mean 3.43 (SD 3.53) during the second wave and mean 1.51 (SD 1.97) postpandemic (see Figure 3). Post hoc Wilcoxon signed-rank tests with Bonferroni correction indicated that all pairwise comparisons were significant (all *P*<.001). This pattern was also reflected in the proportion of VBT sessions relative to total workload (*χ*^2^_2_=52.1, *P*<.001, Kendall *W*=.40, indicating a large effect), decreasing from 29.48% (SD 30.33) to 14.62% (SD 18.13) and 4.32% (SD 5.71) (see Figure 4). The number of patients treated via VBT also decreased significantly from T1 to T2 (*z*=−3.43, *P*<.001, *r*=0.48, indicating a moderate-to-large effect), from mean 8.33 (SD 10.64) to mean 4.45 (SD 6.34) (see Figure 5).

#### Change in Motivations and Barriers

**Figure 6.**
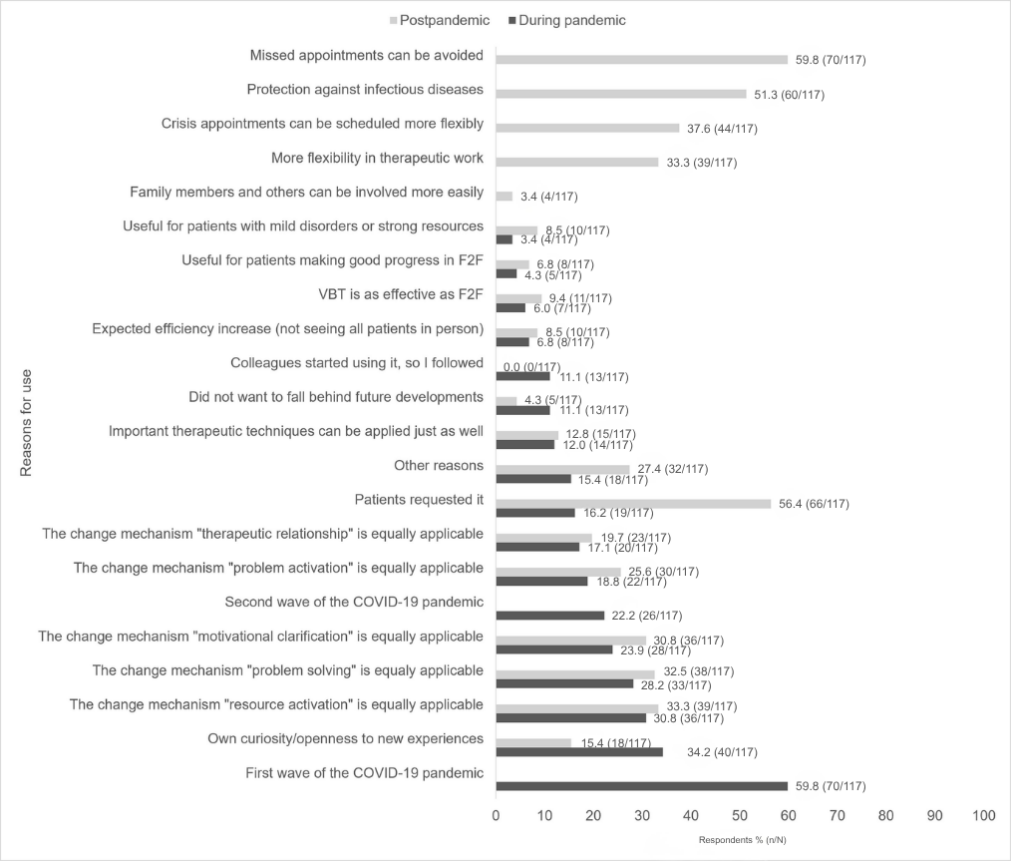
Change in reasons for VBT use. Data were normalized to the sample size. The analyses are based on data of 117 therapists who participated in both survey waves. Answer options “missed appointments can be avoided,” “protection against infectious diseases,” “crisis appointments can be scheduled more flexibly,” “more flexibility in therapeutic work,” and “family members and others can be involved more easily” were added in the second survey. Answer options “first wave of the COVID-19 pandemic” and “second wave of the COVID-19 pandemic” were not included in T2. F2F: face-to-face; VBT: video-based psychotherapy.

Reasons for VBT use shifted from pandemic-specific and exploratory drivers toward more general and practice-related motivations (see Figure 6). Protection against infection was endorsed significantly less often at T2 than in the first (*z*=−3.41, *P*<.001, *r*=0.42) and second pandemic waves (*z*=−3.41, *P*<.001, *r*=0.46), indicating moderate-to-large effects. Exploratory and socially driven motives also declined, including “own curiosity” (*z*=−3.46, *P*<.001, *r*=0.42), “colleagues started using VBT” (*z*=−3.46, *P*<.001, *r*=0.42), and “not wanting to fall behind” (*z*=−2.53, *P*=.01, *r*=0.31), reflecting a small-to-moderate effect. In contrast, patient demand increased significantly from T1 to T2 (*χ*^2^_1_=29.6, *P*<.001, n=67). No significant longitudinal changes were observed for expected efficiency gains, perceived usefulness for specific patients, or the belief that important therapeutic processes could be implemented equally well via VBT (all *P*>.05). Additional T2-only motives were frequently endorsed, particularly avoiding missed appointments (59.8%), protection against infectious diseases (51.3%), more flexible crisis appointments (37.6%), and greater flexibility in therapeutic work (33.3%).

**Figure 7.**
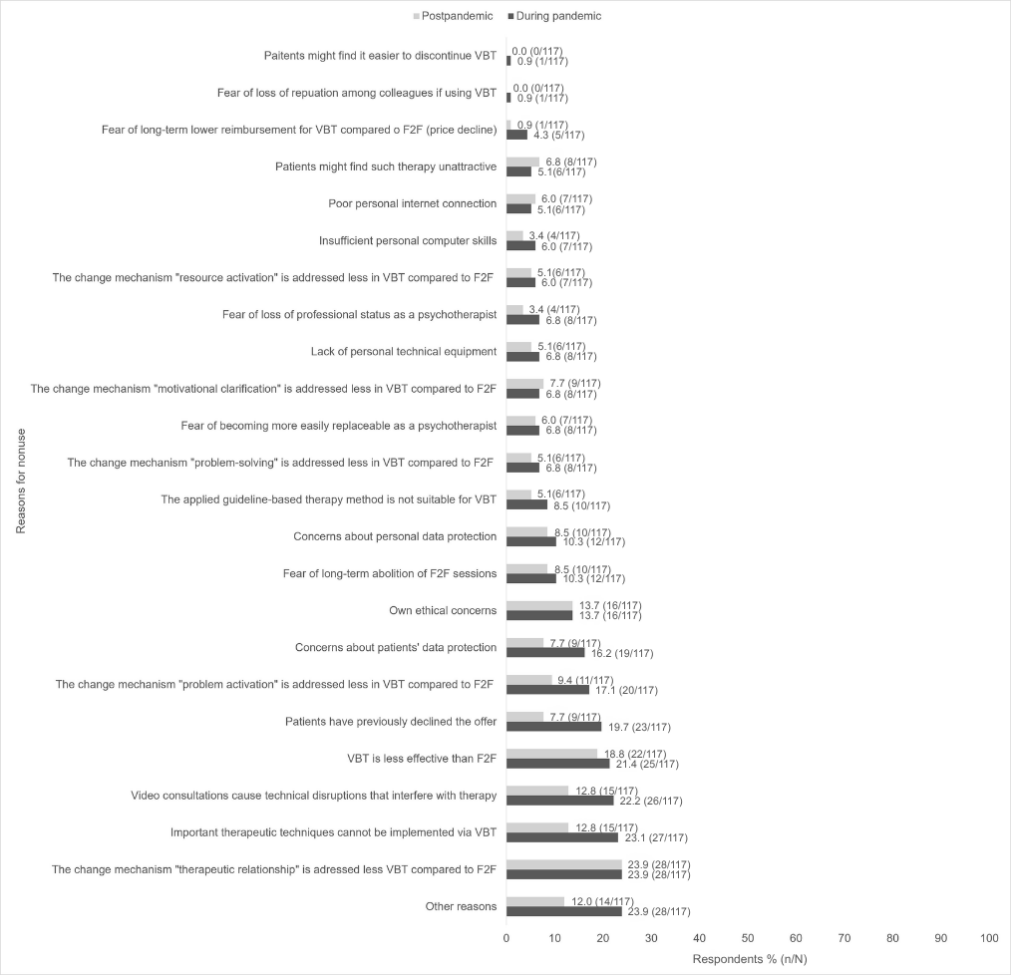
Change in reasons for VBT nonuse**.** Data were normalized to the sample size. The analyses are based on data of 117 therapists who participated in both survey waves. F2F: face-to-face; VBT: video-based psychotherapy.

Perceived barriers to VBT nonuse remained relatively stable over time, although some concerns became less prominent at T2 (see Figure 7). Therapists reported significantly fewer concerns regarding technical disruptions during VBT sessions (*z*=−3.46, *P*<.001, *r*=0.65), difficulties implementing therapeutic techniques via VBT (*z*=−2.32, *P*=.02, *r*=0.44), and reduced opportunities for problem activation (*z*=−2.32, *P*=.02, *r*=0.44). Concerns related to ethical issues, data protection, reduced therapeutic effectiveness, weakened therapeutic relationship, motivational clarification, resource activation, fears of professional replacement, reduced patient progress, therapy discontinuation, or lower patient acceptance did not change significantly over time (all *P*>.05).

#### Perceived Effectiveness and Clinical Evaluations Over Time

Perceived VBT effectiveness did not change significantly over time. Among 110 therapists, 68 (61.8%) reported identical effectiveness ratings at both time points, whereas 27 (24.5%) rated VBT as less effective and 15 (13.6%) as more effective postpandemic. The overall change was not statistically significant (*z*=−1.85, exact *P*=.09, *r*=0.18). Similarly, overall experience with VBT remained stable. A Wilcoxon signed-rank test based on 67 paired observations indicated no significant change between T1 and T2 (*z*=−0.87, exact *P*=.49, *r*=0.11). Descriptively, 17 therapists reported fewer positive experiences over time, 13 reported more positive experiences, and 37 reported no change. Evaluations of VBT’s ability to support Grawe’s therapeutic change mechanisms also remained stable. A McNemar exact test indicated no significant change between time points (exact *P*=.83, n=82), with comparable numbers of therapists changing from “no” to “yes” (n=12) and from “yes” to “no” (n=10).

## Discussion

### Principal Findings

This repeated cross-sectional, partially longitudinal survey study examined postpandemic VBT use among licensed outpatient psychotherapists in Germany. The findings indicate that VBT has become a sustained but selectively used component of outpatient psychotherapy. Use remained above prepandemic levels but declined from pandemic peaks, and motivations shifted from infection-control needs toward practical and patient-centered reasons. Within the UTAUT-T framework, therapy quality expectation was the strongest predictor of behavioral intention, which in turn predicted actual use. Prior VBT experience and regulatory awareness further explained postpandemic use. At the same time, many therapists rated VBT as less effective than F2F therapy and perceived limitations in core therapeutic change mechanisms, particularly the therapeutic relationship. The three identified acceptance profiles further show that postpandemic VBT use is shaped by therapist heterogeneity rather than a simple distinction between users and nonusers.

### Postpandemic VBT Use: Selective Sustainment

The findings suggest that VBT did not disappear after the pandemic but stabilized as a selectively used option rather than becoming a dominant treatment format. This pattern is consistent with international telehealth research showing that digital care remained above prepandemic levels but below pandemic peaks [5,70-74]. The present study extends this trajectory to German outpatient psychotherapy and suggests that postpandemic use is filtered through clinical judgment: therapists retain VBT when it is perceived as practically useful and clinically appropriate but reduce or discontinue it when perceived clinical fit is limited [4,6,43].

This selective sustainment must be understood within the German regulatory context. VBT became billable within statutory health insurance shortly before the pandemic, whereas pandemic-related quota suspensions temporarily facilitated broader use. The return to more regulated use after the pandemic likely contributed to the decline in usage intensity. National billing data and provider studies similarly indicate that reimbursement rules, certified platforms, data protection requirements, and billing conditions shaped VBT uptake and continuation [75,76].

The motivational pattern further supports this interpretation. During the pandemic, VBT was largely driven by external pressure and infection-control needs; postpandemically, patient demand, scheduling flexibility, and avoidance of missed appointments became more relevant [1,6,72,77-79]. However, patient demand does not imply universal suitability, as some patients cannot be reached through VBT or decline this format [80]. Practical barriers such as technical disruptions appear to decrease with experience, whereas broader concerns about effectiveness and therapeutic change mechanisms remain more stable [10,11,34,43,45,81].

### Determinants of Sustained VBT Use: UTAUT-T, Experience, and Regulation

The UTAUT-T findings provide evidence for the psychological determinants of sustained VBT use in a postpandemic context. The model explained a substantial proportion of variance in behavioral intention, and behavioral intention strongly predicted actual postpandemic VBT use, supporting the core UTAUT assumption that intention translates into use behavior [23]. This extends the original UTAUT-T framework by showing that the intention-behavior relationship persists when VBT use is no longer primarily driven by pandemic-related necessity [22]. Because postpandemic use reflects more voluntary clinical decision-making, this link may indicate genuine acceptance rather than situational compliance [72].

Therapy quality expectation emerged as the strongest predictor of behavioral intention, outweighing ease of use, social pressure, and professional support. This is consistent with the original UTAUT-T validation study by Békés et al [22], which conceptualized therapy quality expectation as the psychotherapy-specific equivalent of performance expectancy. In psychotherapy, perceived usefulness refers less to productivity or convenience than to clinical effectiveness and therapeutic quality. Similar findings by Zentner et al [12] and Békés et al [79] further support this interpretation. Sustained VBT integration therefore appears to depend primarily on perceived clinical meaningfulness rather than technical feasibility or convenience.

Ease of use, pressure from others, and professional support also contributed to behavioral intention, although their effects were smaller. This pattern is consistent with UTAUT, where effort expectancy, social influence, and facilitating conditions remain relevant but are often secondary to performance expectancy, particularly after users have gained experience [23]. The nonsignificant role of convenience further suggests that practical advantages alone do not drive VBT acceptance once clinical quality expectations are considered. Nevertheless, professional and social contexts remain important implementation conditions for VBT continuation [12,82].

Beyond behavioral intention, prior pandemic VBT use predicted postpandemic use, supporting a path-dependency interpretation. Pandemic-era exposure may have reduced uncertainty, supported skill development, and helped integrate VBT into professional routines [8,9,73]. However, this may reflect more than habituation: VBT mastery involves clinical competencies associated with therapeutic relationship quality and presence [32]. Regulatory awareness also predicted postpandemic VBT use, although causality remains unclear. Greater awareness may facilitate use or result from use itself, but in either case appears to mark practitioners more embedded in VBT implementation. Given that regulation, reimbursement rules, and unclear guidelines have been identified as recurring barriers to VBT diffusion, regulatory communication should target active users and potential adopters [72].

### Clinical Process Quality: Effectiveness Perceptions and Grawe’s Change Mechanisms

A central finding was the discrepancy between existing outcome evidence and therapists’ clinical process perceptions. Most therapists rated VBT as less effective than F2F therapy, and only about one-third perceived Grawe’s change mechanisms as equally supported. This skepticism is consistent with therapist-reported concerns in Germany and internationally [4,6,16], but contrasts with evidence indicating noninferiority of VBT for symptom reduction, satisfaction, alliance, and dropout across several disorders and treatment contexts [28,29,83-87]. This suggests that sustained VBT integration depends not only on outcome efficacy, but also on perceived process validity [10,32].

This perception-outcome gap may reflect different evaluative frameworks. Outcome studies typically assess symptom change or standardized alliance measures, whereas therapists evaluate moment-to-moment process quality, including therapeutic presence, nonverbal information, relational exchange, and intervention delivery. Prior studies similarly report uncertainty about sensory impressions, eye contact, nonverbal signals, and therapeutic presence in VBT, sometimes with more favorable patient than therapist evaluations [10,32,77,88]. Thus, VBT may not weaken therapeutic processes globally, but it changes the conditions under which they unfold.

This distinction is particularly relevant for Grawe’s change mechanisms. The therapeutic relationship was most often perceived as compromised, followed by problem activation and motivational clarification. These mechanisms rely on emotional engagement, embodied presence, and subtle feedback loops. Qualitative and systematic evidence suggests that VBT changes silence, corporeality, spatial dynamics, and nonverbal interaction, which may complicate the assessment of affective intensity, ambivalence, or highly emotional material [38,84,89-91]. At the same time, alliance and outcome studies show that relational quality can remain stable when therapists and patients adapt to the medium [10,32,84,88,92,93].

More favorable evaluations among VBT users may therefore reflect either acquired mastery or self-selection. Experience may support VBT-specific competencies, but therapists whose style, orientation, or patient groups fit the medium better may also be more likely to continue using it [11,29,32,89]. Because the present design cannot disentangle these mechanisms, implementation should address both possibilities: structured training and supervision are needed, but VBT should also be treated as a modality whose suitability depends on patient characteristics, therapeutic task, therapist competence, and professional fit [94-96].

### Therapist Heterogeneity: Person-Centered Profiles and Regulatory Context

The 3-cluster solution shows that postpandemic VBT acceptance cannot be reduced to a simple user/nonuser distinction. *Tech-savvy optimists* showed high acceptance and the highest actual VBT use, whereas *skeptical traditionalists* showed the lowest acceptance and use and perceived VBT as less able to support core therapeutic change mechanisms. *Cautious neutrals*, the largest group, used VBT more pragmatically and less intensively, suggesting that ambivalence rather than enthusiasm or rejection may characterize the dominant postpandemic stance among German outpatient psychotherapists.

These profiles indicate that sustained VBT implementation is shaped by different configurations of acceptance beliefs rather than by a single linear adoption pathway. Hoffmann’s qualitative typology of German providers offers a conceptual parallel: “pragmatists” viewed VBT as a results-oriented opportunity to improve care, whereas “conservatives” emphasized disruptions to professional routines, responsibilities, and care quality [97]. The present findings therefore suggest that VBT acceptance depends not only on perceived benefit or technological openness, but also on professional identity, therapeutic self-concept, and person-technology fit [97]. Cluster correlates further support this interpretation. *Skeptical traditionalists* tended to be older and less likely to use cognitive behavioral approaches, consistent with prior findings on stronger online-therapy acceptance among cognitive behavioral therapists [6]. The gradient in evaluations of Grawe’s change mechanisms further suggests that VBT acceptance is linked to perceived alliance quality, professional self-doubt, therapeutic presence, and the fit between video settings and therapists’ process orientation [47,98].

The absence of cluster differences in prior VBT training suggests that existing training may not sufficiently address these process-level concerns [32,99]. Prior research indicates that initially skeptical clinicians can become more positive after a supported VBT experience, and that teletherapy mastery involves clinical skills beyond technical proficiency, including compensating for reduced nonverbal information, managing disruptions, and adapting interventions to digital affordances [32,99].

Regulatory perceptions added a profile-specific layer. Regulatory awareness predicted postpandemic VBT use, whereas perceived restrictions were not simple deterrents: *tech-savvy optimists* reported stronger restrictions despite high use, while lower awareness among *skeptical traditionalists* may reflect limited engagement with VBT. Regulatory communication should therefore be profile-sensitive and combine practical clarity with clinically grounded explanations of responsible VBT use [75,76,100,101].

### Implications

The findings have theoretical and practical implications. Theoretically, they support the UTAUT-T as a framework for explaining sustained VBT use beyond crisis-driven adoption [22,23], while also showing that technology acceptance in psychotherapy is not primarily a technical issue. Acceptance depends on whether VBT is perceived as clinically meaningful, therapeutically effective, and compatible with psychotherapeutic work [12,22,32]. The findings also extend technology acceptance models by showing that sustained VBT use is shaped by path-dependent experience [8,9,73] and therapist heterogeneity [47]. Future models of VBT adoption should therefore integrate therapist heterogeneity, professional identity, and perceived clinical process quality more explicitly.

Practically, implementation strategies should move beyond generic technical training. The longitudinal stability of clinical evaluations suggests that passive exposure alone may be insufficient for adaptation, consistent with evidence that teletherapy mastery involves clinical competencies beyond technical familiarity [32,38,88-91]. Training should target VBT-specific clinical competencies, including therapeutic presence, compensation for reduced nonverbal information, intervention adaptation, disruption management, and indication-specific decision-making [32,94-96,99]. Profile-sensitive approaches may be useful: highly accepting therapists may benefit from advanced guidance on indication and quality assurance, ambivalent therapists from case-based supervision, and skeptical therapists from evidence-informed engagement with clinical process concerns [47,97-99]. Therapists may benefit from examining how their own professional orientation and prior experiences shape their perception of VBT, and from distinguishing between concerns grounded in direct experience and concerns based primarily on assumptions [10,32,88,98].

Regulatory and organizational guidance should be clear, stable, and clinically actionable. Information about billing, certified platforms, data protection, quality standards, and appropriate indications should reach both active users and potential adopters [72,75,76,100,101]. Because VBT may not fit all therapeutic approaches, patient groups, or professional identities equally well [11,80,91,97,98], sustainable implementation should aim for flexible, evidence-informed, and clinically differentiated use rather than universal digital transformation. In practice, this requires shared decision-making that integrates empirical evidence, clinical judgment, and patient preferences [102,103].

### Limitations

Several limitations should be considered when interpreting these findings. First, the postal survey design with voluntary participation may have introduced self-selection bias, potentially overrepresenting psychotherapists with a greater interest in or experience with VBT.

Second, attrition in the longitudinal subsample may have biased longitudinal findings. Participants who completed both waves were younger and reported more positive VBT experiences at T1 than dropouts, potentially leading to an overestimation of positive trajectories. The longitudinal subsample was too small for more advanced modeling approaches, such as growth curve models or latent transition analyses.

Third, all outcomes were based on therapist self-report and were not validated against objective data. Patient outcomes, patient preferences, session recordings, behavioral observations, or standardized process measures were not assessed. Thus, therapists’ perceptions of VBT effectiveness and process quality may not correspond to patients’ experiences or objective outcomes.

Fourth, measurement limitations should be noted. Although the UTAUT-T is a validated instrument [22,23], some subscales showed limited internal consistency in the present sample, which may have weakened associations and affected regression and cluster-based findings. In addition, measures of perceived effectiveness and Grawe’s change mechanisms were developed for this study, limiting comparability with prior research and underscoring the need for validated instruments assessing clinical process quality in VBT.

Fifth, the cluster analysis was exploratory. The profiles depended on the selected UTAUT-T variables, and cluster validity indices indicated only moderate separation. Although the 2-cluster solution showed somewhat better internal separation, the 3-cluster solution was retained because it provided greater theoretical and clinical interpretability by identifying an ambivalent group between high acceptance and skepticism. This group reflected heterogeneity beyond a simple user versus nonuser distinction. The profiles should be understood as exploratory acceptance patterns rather than sharply separated natural groups and require replication in independent samples.

Sixth, the predominantly cross-sectional design limits causal inference. Although the longitudinal subsample allowed examination of within-person change, the study cannot determine whether attitudes drive behavior, behavior shapes attitudes, experience leads to adaptation, or self-selection explains user-nonuser differences. Similarly, regulatory awareness may either facilitate VBT use or result from using VBT.

Finally, the sample was restricted to psychotherapists in East German federal states, was predominantly female, and was largely cognitive behavioral therapy oriented. Findings may therefore not generalize to other countries with different health care and reimbursement systems, other therapeutic orientations, or more gender-balanced samples. The results should be interpreted within the specific context of postpandemic German outpatient psychotherapy and its regulatory conditions.

### Future Research

Future research should examine VBT as a clinically differentiated modality rather than as a uniform alternative to F2F therapy. Qualitative studies are needed to identify how psychotherapists adapt therapeutic techniques and clinical decision-making to VBT, while longitudinal studies should clarify whether acceptance profiles are stable orientations or change with experience, training, and regulation. Patient outcomes, preferences, satisfaction, and perceived process quality should be assessed with validated instruments. Finally, intervention studies should test whether competency-based VBT training improves acceptance, therapeutic presence, and clinical decision-making more effectively than generic technical training.

### Conclusions

This study extends existing pandemic-era and cross-sectional VBT research by examining how technology acceptance, clinical process quality, and therapist heterogeneity shape postpandemic VBT sustainability under more voluntary routine-care conditions. This approach differs from prior studies that primarily focused on pandemic use or cross-sectional acceptance. The findings show that VBT has become a sustained but selectively used component of German outpatient psychotherapy rather than evidence of a comprehensive digital transformation or a universal replacement for F2F care.

The study contributes to the field by showing that sustained VBT use depends on perceived clinical meaningfulness, prior experience, regulatory familiarity, and therapist acceptance profiles. The identified heterogeneity indicates that implementation strategies should not assume a simple user versus nonuser distinction. Instead, VBT should be understood as a flexible modality whose value depends on indication, patient preference, therapeutic task, therapist competence, and organizational conditions.

In real-world practice, sustainable VBT implementation requires clinically differentiated and evidence-informed use. This includes profile-sensitive training, clear regulatory and organizational guidance, and shared decision-making that integrates empirical evidence, clinical judgment, and patient preferences. Further research should examine clinical process quality and identify when, for whom, and under which conditions VBT provides added value within routine outpatient psychotherapy.

## Supplementary material

10.2196/82972 Multimedia Appendix 1 Overview of measures and operationalization.

10.2196/82972 Multimedia Appendix 2 Questionnaire.

10.2196/82972 Multimedia Appendix 3 Supplementary tables on internal consistency, attrition, and between-cluster differences.

10.2196/82972 Checklist 1 STROBE checklist.

## Abbreviations

F2F: face-to-face
OPK: Ostdeutsche Psychotherapeutenkammer
OR: odds ratio
STROBE: Strengthening the Reporting of Observational Studies in Epidemiology
UTAUT-T: unified theory of acceptance and use of technology for therapists
VBT: video-based psychotherapy
