# Supplementary material for: Postpandemic Use of Video-Based Psychotherapy Among German Outpatient Psychotherapists: Repeated Cross-Sectional and Partially Longitudinal Survey Study
Source: J Med Internet Res. 2026 Jul 31;28:e82972. doi: 10.2196/82972 (PMC13426897; doi:10.2196/82972)
Supplement: Multimedia Appendix 1 [file jmir-v28-e82972-s001.docx]

**Multimedia Appendix 1:** Overview of measures and operationalization.

| **Construct** |  | **Measurement Type** | **No. of**  **Items** | **Response Format** | **Operationalization / Scoring** | **Example Item/ Description** | **Role in Analyses** | **Source** |
| --- | --- | --- | --- | --- | --- | --- | --- | --- |
| **Post-pandemic VBT Use** | | | | | | | | |
| **Post-pandemic VBT usage status** |  | Derived variable | - | Derived (dichotomous) | Classified as user if ≥5 sessions with  ≥3 patients | Based on the self-reported number of VBT sessions and treated patients | Primary outcome, grouping variable, between-cluster comparisons | Adapted from Leukhardt et al. (2021) [11] |
| **Post-pandemic VBT use (any use)** |  | Study-specific item | 1 | Dichotomous (yes/no) | Having used VBT at least once (yes) or not (no) | Have you used VBT at least once since April 7, 2023 (lifting of COVID-19 restrictions)? | filter item | Study-specific |
| **Post-pandemic VBT usage intensity (number of sessions)** |  | Study-specific item | 1 | Open-ended (numeric) | Estimated total number of VBT sessions conducted since April 7, 2023 (lifting of COVID-19 restrictions); continuous variable; contributed to the operationalization of post-pandemic VBT usage status (user vs non-user) | How many VBT sessions have you conducted in total since April 7, 2023 (lifting of COVID-19 restrictions)? | usage intensity between-cluster comparisons, longitudinal usage change | Study-specific |
| **Post-pandemic VBT usage intensity (number of patients)** |  | Study-specific item | 1 | Open-ended (numeric) | Estimated number of different patients treated via VBT since April 7, 2023 (lifting of COVID-19 restrictions); continuous variable; contributed to the operationalization of post-pandemic VBT usage status (user vs non-user) | How many different patients have you treated via VBT since April 7, 2023 (lifting of COVID-19 restrictions)? | usage intensity between-cluster comparisons, longitudinal usage change | Study-specific |
| **Post-pandemic VBT usage intensity (weekly frequency)** |  | Study-specific item | 1 | Open-ended (numeric) | estimated average number of VBT sessions conducted per week since April 7, 2023 (lifting of COVID-19 restrictions); continuous variable | How many VBT sessions have you conducted on average per week since April 7, 2023 (lifting of COVID-19 restrictions)? | usage intensity between-cluster comparisons, longitudinal usage change | Study-specific |
| **Relative VBT use (proportion of total workload)** |  | Study-specific item | 1 | Open-ended (percentage, 0-100) | estimated percentage of VBT sessions relative to their total weekly workload since April 7, 2023 (lifting of COVID-19 restrictions); continuous variable | What percentage of your weekly workload has been conducted via VBT since April 7, 2023 (lifting of COVID-19 restrictions)? | usage intensity, dependent variable in regression, between-cluster comparisons, longitudinal usage change | Study-specific |
| **Retrospective Use** | | | | | | | | |
| **Pre-pandemic VBT use** |  | Study-specific item | 1 (plus optional date entry) | Categorical (three response options) with optional open-ended date field | Classified as users (≥5 VBT sessions with ≥3 patients), non-users, or not working in outpatient care during the reference period | Did you use video-based psychotherapy before the COVID-19 pandemic (at least 5 sessions with at least 3 different patients)? | usage comparisons | Adapted from Leukhardt et al. (2021) [11] |
| **COVID-19 pandemic VBT use** |  | Study-specific item | 1 | Categorical (three response options) | Classified as users (≥5 VBT sessions with ≥3 patients), non-users, or not working in outpatient care during the reference period | Did you use VBT during the COVID-19 pandemic (at least 5 sessions with at least 3 different patients)? | usage comparisons, predictor, between-cluster comparisons | Adapted from Leukhardt et al. (2021) [11] |
| **Technology Acceptance (UTAUT-T)** | | | | | | | | |
| **Behavioral intention of VBT use** |  | Validated scale (German translation of the UTAUT-T therapist version) | 2 | Likert scale (1–5) | Mean score; higher score = stronger intention to use VBT | I intend to use VBT after the end of the pandemic. | dependent variable in regression analyses, correlation, cluster variable   \|  \| \| --- \|  \|  \| \| --- \| | Békés et al. (2022) [22] |
| **Therapy Quality Expectation** |  | Validated scale (German translation of the UTAUT-T therapist version) | 9 | Likert scale (1–5) | Mean score; reverse-coded items were recoded; higher scores = more positive expectations regarding therapy quality | The quality of VBT is the same as F2F therapy. | predictor regression analyses, correlation, cluster variable | Békés et al. (2022) [22] |
| **Ease of use** |  | Validated scale (German translation of the UTAUT-T therapist version) | 4 | Likert scale (1–5) | Mean score; reverse-coded items were recoded; higher scores = greater perceived ease of use | I find providing VBT easy. | predictor regression analyses, correlation, cluster variable | Békés et al. (2022) [22] |
| **Pressure from others** |  | Validated scale (German translation of the UTAUT-T therapist version) | 2 | Likert scale (1–5) | Mean score; higher scores = stronger perceived social pressure to use VBT | People who are important to me think that I should use VBT. | predictor regression analyses, correlation, cluster variable | Békés et al. (2022) [22] |
| **Professional support** |  | Validated scale (German translation of the UTAUT-T therapist version) | 2 | Likert scale (1–5) | Mean score; higher scores = greater perceived support | My professional organization supports VBT. | predictor regression analyses, correlation, cluster variable | Békés et al. (2022) [22] |
| **Convenience** |  | Validated scale (German translation of the UTAUT-T therapist version) | 2 | Likert scale (1–5) | Mean score; higher scores = greater perceived convenience | Using VBT saves me time and/or money. | predictor regression analyses, correlation | Békés et al. (2022) [22] |
| **Clinical Process Quality** | | | | | | | | |
| **Perceived effectiveness of VBT vs. F2F** |  | Study-specific item | 1 | Ordinal (5-point Likert scale) | Higher values = lower perceived effectiveness of VBT | How would you rate the effectiveness of VBT compared to F2F sessions? | Secondary outcome, predictor regression analyses,  user-non-user comparisons, longitudinal change | Study-specific |
| **Overall equivalence of change mechanisms (Grawe)** |  | Study-specific item | 1 global item + 5 change mechanisms assessed conditionally | Dichotomous (yes/no) followed by multiple selection (dichotomous per mechanism) | First: whether change mechanisms are addressed comparably to F2F therapy (yes/no); if no, specified which mechanisms less or more addressed; each mechanism coded as a separate dichotomous variable (0 = not selected, 1 = selected); no total score | Are the five therapeutic change mechanisms according to Grawe addressed in VBT in a comparable way to F2F psychotherapy? | Secondary outcome, predictor regression analyses,  user-non-user comparisons, between-cluster comparisons, longitudinal change | Study-specific |
| **Experience with VBT** | | | | | | | | |
| **Overall experience with VBT** |  | Study-specific item | 1 | Ordinal (5-point Likert scale) | Higher scores = more negative experiences with VBT (1 = very positive, 5 = very negative) | How would you rate your overall experience with VBT? | Descriptive, Attrition, user-non-user comparisons, longitudinal change | Study-specific |
| **Detailed experiences with VBT** |  | Study-specific item | 1 | Multiple selection (dichotomous per option) | Each option coded separately (0/1); no total score | How would you describe your experiences with VBT? | Descriptive, Attrition, longitudinal change | Study-specific |
| **Challenges in conducting VBT** |  | Study-specific item | Multiple | Multiple selection (dichotomous per item) | Each challenge coded separately (0/1); no total score | What challenges do you face when conducting VBT? | Descriptive/ exploratory |  |
| **Motivations and Barriers** | | | | | | | | |
| **Reasons for VBT use** |  | Study-specific item | Multiple | Multiple selection (dichotomous per item) | Each reason coded separately (0/1); no total score | What motivates you to continue using VBT? | Descriptive/ exploratory,  longitudinal change | Study-specific |
| **Reasons for VBT non-use** |  | Study-specific item | 1 initial dichotomous item + multiple barrier items | Dichotomous (yes/no) followed by multiple selection (dichotomous per barrier) | Each barrier coded separately (0/1); no total score | Have you used VBT after the lifting of COVID-19 restrictions? (If no: What are the reasons? | Descriptive/ exploratory,  longitudinal change | Study-specific |
| **Digital Affinity** | | | | | | | | |
| **Private internet use** |  | Study-specific item | 1 | Ordinal (6-point Likert scale) | Higher scores = lower frequency of private internet use (1 = hourly, 6 = never) | How often do you use the internet privately (on smartphone, tablet, PC, or laptop)? | Descriptive, Attrition, user-non-user comparisons | Study-specific |
| **Professional internet use** |  | Study-specific item | 1 | Ordinal (5-point Likert scale) | Higher scores = lower professional internet use relative to colleagues (1 = much more, 5 = much less) | How much do you use the internet for your professional work compared to your colleagues? | Descriptive, Attrition, predictor regression analyses, user-non-user comparisons, between-cluster comparisons | Study-specific |
| **Regulatory Variables** | | | | | | | | |
| **Awareness of VBT regulations** |  | Study-specific item | 1 | Ordinal (4-point Likert scale) | Higher = less aware | I am familiar with the regulations regarding the use of VBT. | predictor regression analyses, between-cluster comparisons | Study-specific |
| **Perceived restrictions** |  | Study-specific item | 1 | Ordinal (4-point Likert scale) | Higher = less perceived restriction | The regulations regarding the use of video-based psychotherapy restrict me. | predictor regression analyses, between-cluster comparisons | Study-specific |
| **Sociodemographic and Professional Variables** | | | | | | | | |
| **Age** |  | Study-specific item | 1 | Numeric | Age in years (continuous variable) | How old are you? ___ years | Descriptive, Attrition, predictor regression analyses, between-cluster comparisons | Study-specific |
| **Gender** |  | Study-specific item | 1 | Nominal | three response options (male, female, diverse) | Please select your gender: male, female, diverse | Descriptive, Attrition, | Study-specific |
| **Therapeutic approach** |  | Study-specific item | 1 | Nominal | Dichotomous coding for each therapeutic approach (0 = not selected, 1 = selected) | Which therapeutic approaches do you practice?: cognitive behavioral, psychodynamic, psychoanalytic, systemic | Descriptive, Attrition, predictor regression analyses, user-non-user comparisons, between-cluster comparisons | Study-specific |
| **Professional experience** |  | Study-specific item | 1 | Numeric | Years working as a licensed psychotherapist (continuous variable) | How many years have you been working as a psychotherapist? ___ years | Descriptive, Attrition | Study-specific |
| **Professional experience in outpatient practice** |  | Study-specific item | 1 | Numeric | Years working in outpatient practice (continuous variable) | For how many years have you been working in outpatient psychotherapeutic care? ___ years | Descriptive, Attrition | Study-specific |
| **Residential location (urbanicity)** |  | Study-specific item | 1 | Ordinal | Categorical variable with four levels based on population size | Do you live in a rural area ≤5.000; small town 5.000–20.000; medium-sized town 20.000–100.000; large city >100.000 inhabitants | Descriptive | Study-specific |
| **Practice location (federal state)** |  | Study-specific item | 1 | Nominal | Categorical variable indicating federal state of practice | In which federal state do you practice as an outpatient psychotherapist? (Brandenburg, Mecklenburg-Western Pomerania, Saxony, Saxony-Anhalt, Thuringia) | Descriptive | Study-specific |
| **Workload** |  | Study-specific item | 1 | Ordinal | Categorical variable indicating contractual care provision level | What is your contractual care provision level? (50%, 75%, 100%) | Descriptive | Study-specific |
| **Additional Measures / Contextual Variables** | | | | | | | | |
| **Prior VBT training** |  | Study-specific item | 1 | Categorical (dichotomous) | Dichotomous variable (0 = no, 1 = yes) | Have you attended any training on VBT? | between-cluster comparisons | Study-specific |

**Note.** All measures were based on self-report.
