# Supplementary material for: Postpandemic Use of Video-Based Psychotherapy Among German Outpatient Psychotherapists: Repeated Cross-Sectional and Partially Longitudinal Survey Study
Source: J Med Internet Res. 2026 Jul 31;28:e82972. doi: 10.2196/82972 (PMC13426897; doi:10.2196/82972)
Supplement: Multimedia Appendix 2 [file jmir-v28-e82972-s002.pdf]

Therapeut*innen-Befragung

Sehr geehrte Teilnehmer*innen,

der vorliegende Fragebogen ist Teil des Forschungsprojekts „Videobasierte ambulante Psychotherapie“. In Zusammenarbeit mit der AOK Sachsen-Anhalt wollen wir untersuchen, ob und wie niedergelassene approbierte Psychotherapeut*innen die Videosprechstunde nach Aufhebung der Pandemiebeschränkungen in ihre Behandlungen einbinden und wie sich der Stellenwert der Videosprechstunde entwickelt hat. Unter "Videosprechstunde" verstehen wir alle per zertifizierter Videokonferenz möglichen Leistungen der Richtlinien-Psychotherapie.

Bitte beantworten Sie die Fragen ehrlich, indem Sie Ihre persönliche Präferenz angeben, ohne zu ausgiebig zu überlegen. Es gibt keine richtigen oder falschen Antworten.

Die Umfrage erfolgt in pseudonymisierter Form (selbst generierter persönlicher Code) Dieser Code würde es erlauben, die Daten der ersten Erhebungswelle 2020 (falls Sie daran teilgenommen haben), sowie die Daten einer eventuellen Folgeerhebung mit der jetzigen Erhebung zu verbinden und so wertvolle Längsschnittdaten zu gewinnen. Ein Rückschluss auf Ihre Identität ist für uns aufgrund Ihres selbst kreierten Codes jedoch nicht möglich.

Ihre Angaben werden selbstverständlich streng vertraulich behandelt, nicht an Dritte weitergegeben und lediglich für wissenschaftliche Forschungszwecke genutzt (siehe beigefügte Datenschutzerklärung).

Die Beantwortung der Umfrage wird in etwa 20-30 Minuten Ihrer Zeit in Anspruch nehmen. Bitte beachten Sie die Anweisungen im Fragebogen, wie die Sprungregeln zwischen den Fragen.

Bitte senden Sie uns, den Fragebogen nach Kenntnisnahme der Einwilligungserklärung im beiliegenden großen Briefumschlag und die separate schriftliche Einwilligungserklärung (Interviewteilnahme, follow-up Studie, Ergebnisrückmeldung und Teilnahme an der Verlosung der Dankeschön-Preise) im beiliegenden kleinen Briefumschlag bis 03.05.24 (Poststempel) zu.

Bei Fragen, die die Studienleitung betreffen, können Sie sich gern an Herrn Prof. Dr. Spaeth (m.spaeth@meu.de) wenden.

Codierung

In den folgenden Feldern soll eine ***Pseudonymisierung*** *Ihrer persönlichen Daten erfolgen, damit die Daten der ersten Erhebungswelle mit der jetzigen zweiten Erhebung verbunden werden können und damit Sie bei einer möglichen follow-up Erhebung, mit Ihrem Einverständnis, nochmals kontaktiert werden können.*

Bitte geben Sie hier zur besseren Codierung den <u>ersten und letzten</u> Buchstaben des Vornamens Ihrer Mutter ein. (z.B. Julia = JA)

Bitte geben Sie hier zur besseren Codierung den <u>ersten und letzten</u> Buchstaben des Vornamens Ihres Vaters ein. (z.B. Jan = JN)

Bitte geben Sie zur besseren Codierung den <u>ersten und letzten</u> Buchstaben Ihres Vornamens ein. (z.B. Sabine = SE)

Bitte geben Sie zur besseren Codierung den <u>Tag</u> des Geburtsdatums Ihrer Mutter ein. (z.B. 21. Mai 1960 = 21)

I. Demographische Daten

Bitte beziehen Sie sich bei den folgenden Fragen auf Ihre derzeitige Tätigkeit im ambulanten psychotherapeutischen Bereich.
*Sollten Sie eine Doppelapprobation (Psychologische*r Psychotherapeut*in* ***sowie*** *Kinder- und Jugendlichenpsychotherapeut*in) besitzen, beziehen Sie sich bitte bei der Beantwortung Ihres Fragebogens, nur auf die* ***Behandlung von erwachsenen Patient*innen*** *(ab 18 Jahren)!*

I.1. Bitte wählen Sie Ihr Geschlecht aus.

☐ männlich
☐ weiblich
☐ divers

I.2. Wie alt sind Sie?

____ Jahre

I.3. Leben Sie im/in der

☐ ländlichen Raum (bis 5000 Einwohner)
☐ Kleinstadt (5000 – 20000 Einwohner)
☐ Mittelstadt (20000 – 100000 Einwohner)
☐ Großstadt (mehr als 1000000 Einwohner)

I.4. In welchem Bundesland praktizieren Sie als niedergelassene*r Psychotherapeut*in?

☐ Brandenburg
☐ Mecklenburg-Vorpommern
☐ Sachsen
☐ Sachsen-Anhalt
☐ Thüringen

I.5. Welche Approbation haben Sie? *(Mehrfachantworten möglich)*

☐ Psychologische*r Psychotherapeut*in
☐ Kinder- und Jugendlichenpsychotherapeut*in

I.6. Welches Richtlinienverfahren üben Sie aus? *(Mehrfachantworten möglich)*

☐ Verhaltenstherapie
☐ Tiefenpsychologisch-psychodynamisch
☐ Psychoanalytisch
☐ Systemisch

I.7. Wie viele Jahre sind Sie bereits tätig als Psychologische*r Psychotherapeut*in?

_____ Jahre

I.8. Seit wie vielen Jahren sind Sie in ambulanter psychotherapeutischer Praxis tätig?

_____ Jahre

I.9. In welcher Form üben Sie Ihre vertragsärztliche ambulante psychotherapeutische Tätigkeit aus?

- ☐ selbständig mit Kassenzulassung
- ☐ im Jobsharing
- ☐ angestellt

I.10. Wie hoch ist Ihr Versorgungsauftrag?

- ☐ 100%
- ☐ 75%
- ☐ 50%

II. Private Mediennutzug

*Im Folgenden erfragen wir Ihr Verhalten in Bezug auf die **private** Mediennutzung.*

II.1. Welche der folgenden Geräte und Mediendienste nutzen Sie privat im Alltag? *(Mehrfachantworten möglich)*

- ☐ Smartphone
- ☐ Tablet
- ☐ PC oder Notebook
- ☐ E-Mail
- ☐ Internetbrowser (surfen und/oder einkaufen)
- ☐ App-Nutzung (auf Smartphone, Tablet, PC, Laptop)
- ☐ Videoportale (YouTube, Twitch, Vevo, Vimeo usw.)
- ☐ Social Media (Facebook, Instagram, Twitter/X usw.)
- ☐ Messenger-Dienste (WhatsApp, Telegram, Threema, Signal usw.)
- ☐ Skype
- ☐ Videokonferenzsysteme (Zoom, Microsoft Teams usw.)
- ☐ Streamingdienste (Netflix, Amazon Prime, Disney+, Sky Ticket usw.)
- ☐ Online-Zeitungen und Zeitschriften
- ☐ Online-Musik, Videos, Filme, Podcasts
- ☐ Künstliche Intelligenz (z.B. Chat GPT)

II.2. Wie häufig benutzen Sie das Internet privat (auf Smartphone, Tablet, PC oder Laptop)?

- ☐ stündlich
- ☐ mehrfach am Tag
- ☐ täglich
- ☐ wöchentlich
- ☐ monatlich
- ☐ nie

II.3. Wie würden Sie Ihren Umgang mit dem Internet beschreiben?

- ☐ sehr sicher
- ☐ sicher
- ☐ es geht
- ☐ unsicher
- ☐ sehr unsicher

II.4. Wie würden Sie Ihren Umgang mit Videokonferenzsystemen beschreiben (Zoom, Microsoft Teams, WebEx, Google Meet usw.)?

- ☐ sehr sicher
- ☐ sicher
- ☐ es geht
- ☐ unsicher
- ☐ sehr unsicher

III. Mediennutzung in der psychotherapeutischen Praxis

Im Folgenden erfragen wir Ihr Verhalten in Bezug auf die Mediennutzung im Rahmen Ihrer ***ambulanten psychotherapeutischen Tätigkeit****.*

III.1. Wie stark nutzen Sie das Internet für Ihren Beruf als Psychotherapeut*in (z.B. Therapiematerial, Recherche, eigene Fortbildungen, therapeutische Aufgaben für die Patient*innen, Organisation der Praxis)?

- ☐ viel mehr als meine Kolleg*innen
- ☐ mehr als meine Kolleg*innen
- ☐ durchschnittlich
- ☐ weniger als meine Kolleg*innen
- ☐ viel weniger als meine Kolleg*innen

III.2. Verfügen Sie über eine Praxishomepage?

- ☐ ja
- ☐ nein

III.3. Welche Medien und Dienste haben Sie <u>bereits jemals</u> in der psychotherapeutischen Behandlung von Patient*innen verwendet?
(Mehrfachantworten möglich)

- ☐ Telefon
- ☐ SMS
- ☐ E-Mail
- ☐ Messengerdienste (WhatsApp, Telegram, Threema, Signal usw.)
- ☐ Videoportale (YouTube, Twitch, Vevo, Vimeo usw.)
- ☐ Medizinisch-psychologische Apps (gegen Depression, Ängste etc.)
- ☐ Skype
- ☐ Videokonferenzsysteme (Zoom, Microsoft Teams usw.)
- ☐ Social Media Dienste (Facebook, Instagram, Twitter/X usw.)
- ☐ Online-Informationssuche (Google, Yahoo usw.)
- ☐ Künstliche Intelligenz (z.B. Chat GPT)

IV. Nutzung der Videosprechstunde

*Folgende Fragen beziehen sich auf die Nutzung der Videosprechstunde im **Einzelsetting**.*

IV.1. Die fünf Wirkfaktoren der Psychotherapie nach Klaus Grawe werden in der Videosprechstunde vergleichbar zur Präsenzpsychotherapie angesprochen:

☐ ja

☐ nein, da folgende Wirkfaktoren in der Videosprechstunde weniger angesprochen werden *(kreuzen Sie eine oder mehrere der folgenden Wirkfaktoren an):*

- o Problemaktualisierung (unmittelbare Erfahrung)
- o Problembewältigung
- o Therapeutische Beziehung
- o Ressourcenarbeit
- o Motivationale Klärung

☐ nein, da folgende Wirkfaktoren in der Videosprechstunde mehr angesprochen werden *(kreuzen Sie eine oder mehrere der folgenden Wirkfaktoren an):*

- o Problemaktualisierung (unmittelbare Erfahrung)
- o Problembewältigung
- o Therapeutische Beziehung
- o Ressourcenarbeit
- o Motivationale Klärung

IV.2. Haben Sie während der Pandemiebeschränkungen im Rahmen der Covid-19-Pandemie (16.03.2020 – 07.04.2023) das Angebot der Videosprechstunde genutzt, d.h. insgesamt mind. 5 Videosprechstunden bei wenigstens 3 verschiedenen Patient*innen durchgeführt?

☐ ja

☐ nein

☐ ich war zu diesem Zeitpunkt noch nicht ambulant als Psychotherapeut*in tätig

IV.3. Haben Sie bereits vor Beginn der ersten Covid-19-Schutzmaßnahmen (Stichtag: 16.03.2020) das Angebot der Videosprechstunde genutzt, d.h. insgesamt mind. 5 Videosprechstunden bei wenigstens 3 verschiedenen Patient*innen durchgeführt?

☐ ja, ungefähr seit ______________ (Bitte Angabe des Datums)

☐ nein

☐ ich war zu diesem Zeitpunkt noch nicht ambulant als Psychotherapeut*in tätig

IV.4. Haben Sie nach Ende der Pandemiebeschränkungen im Rahmen der Covid-19-Pandemie (<u>seit 07.04.2023</u>) das Angebot der Videosprechstunde <u>mindestens einmal</u> genutzt?

□ ja

□ nein, weil *(Mehrfachantworten möglich)*:

- o a) Patient*innen lehnten bisher das Angebot einer Videosprechstunde ab
- o b) Patient*innen könnten während einer Therapie mit Videosprechstunde leichter die Therapie beenden
- o c) Patient*innen könnten eine solche Therapie unattraktiv finden
- o d) eigene ethische Bedenken
- o e) zu wenig eigene Computerkenntnisse
- o f) eigene technische Ausstattung fehlt
- o g) eigene schlechte Internetverbindung
- o h) Befürchtungen bzgl. eigenem Datenschutz
- o i) Befürchtungen bzgl. Datenschutz der Patient*innen
- o j) befürchtete langfristige Abschaffung von Präsenzstunden
- o k) langfristig befürchtete mindere Bezahlung der Videosprechstunde im Vergleich zur Präsenzpsychotherapie (Preisverfall)
- o l) befürchteter Statusverlust als Psychotherapeut*in
- o m) befürchtete leichtere Ersetzbarkeit als Psychotherapeut*in
- o n) befürchteter Imageverlust bei Kolleg*innen wenn Videosprechstunde eingesetzt wird
- o o) Videosprechstunde verursacht technische Störungen, die wiederum die Therapie stören
- o p) wichtige therapeutische Techniken lassen sich mit der Videosprechstunde nicht umsetzen. Welche?:____
  ____
- o q) das eingesetzte Richtlinienverfahren passt nicht zur Videosprechstunde
- o r) Videosprechstunde ist weniger wirksam als Präsenzpsychotherapie

Wirkfaktoren nach Grave werden in der Videosprechstunde weniger angesprochen als in der Präsenzpsychotherapie:

- o s) Problemaktualisierung (unmittelbare Erfahrung)
- o t) Problembewältigung
- o u) Therapeutische Beziehung
- o v) Ressourcenarbeit
- o w) Motivationale Klärung

- o x) sonstige Gründe: ____
  ____

Welches sind die Ihnen wichtigsten Argumente (a-x)? Bis zu 3 Nennungen möglich. Bitte geben sie die Buchstaben der Argumente an und/oder ihre eigenen Argumente aus „x)“: ____

____

*Wenn die letzte Frage (IV.4.) mit **JA** beantwortet haben, machen Sie bitte gleich mit **Frage Nr. IV.5.** weiter!*

*Wenn Sie die letzte Frage (IV.4.) mit **NEIN** beantwortet haben, machen Sie bitte mit **Frage Nr. IV.19.** auf Seite 13 weiter!*

IV.5. Was bewegt Sie dazu, auch nach der Aufhebung der Covid-19-Pandemiebeschränkungen die Videosprechstunde weiterhin einzusetzen? *(Mehrfachantworten möglich)*

☐ a) Patient*innen haben danach gefragt

☐ b) eigene Neugier/Offenheit für neue Erfahrungen

☐ c) erhoffte Effizienzsteigerung meinerseits, da man nicht alle Patient*innen real sehen muss

☐ d) sinnvoll, da einige Patient*innen so eine geringe Störung haben bzw. so ressourcenreich sind, dass ich sie nicht in Präsenz sehen muss

☐ e) sinnvoll, da einige Patient*innen so gute Fortschritte während der Präsenztherapie machen, dass ich sie irgendwann nicht mehr in Präsenz sehen muss

☐ f) Schutz vor Infektionskrankheiten, wenn Patient*in oder ich zeitweise ansteckend sind

☐ g) Kolleg*innen haben damit angefangen und ich zog nach

☐ h) ich wollte nicht den Anschluss an die Zukunft verlieren

☐ i) wichtige therapeutische Techniken lassen sich mit der Videosprechstunde genauso umsetzen wie in der Präsenzpsychotherapie:
Welche?: ____
____

☐ j) Videosprechstunde ist genauso wirksam wie Präsenzpsychotherapie

☐ k) Familienangehörige und andere Personen können leichter in die Therapie einbezogen werden

☐ l) ich bin in meiner therapeutischen Arbeit flexibler

☐ m) Krisentermine können flexibler angeboten werden

☐ n) Terminausfälle können vermieden werden (z.B. bei kurzfristigen Erkrankungen, Zugausfällen etc.)

Wirkfaktoren nach Grawe lassen sich genauso gut umsetzen wie in der Präsenzpsychotherapie:

☐ o) Problemaktualisierung (unmittelbare Erfahrung)

☐ p) Problembewältigung

☐ q) Therapeutische Beziehung

☐ r) Ressourcenarbeit

☐ s) Motivationale Klärung

☐ t) sonstige Gründe: ____
____

Welches sind die Ihnen wichtigsten Argumente (a-t)? Bis zu drei Nennungen möglich. Bitte geben Sie die Buchstaben der Argumente an und/oder Ihre eigenen Argumente aus „t)“ an: ____
____

IV.6. Wie waren Ihre Erfahrungen mit der Videosprechstunde bisher?

☐ sehr positiv

☐ positiv

☐ gemischt

☐ negativ

☐ sehr negativ

IV.7. Welche Anforderungen der Videosprechstunde nehmen Sie als belastend wahr? *(Mehrfachantworten möglich)*

☐ einen erhöhten Zeitaufwand

☐ eigene Unsicherheiten und Ängste

☐ Anschaffung neuer Materialien (Technikzubehör, Headset, Laptop, Webcam usw.)

☐ Einlesen in die Regularien z.B. von der KBV

☐ Organisation eines zertifizierten Anbieters für die Videosprechstunde

☐ finanzieller Aufwand

☐ eine andere, nämlich: ____

IV.8. Wie viele Videosprechstunden haben Sie insgesamt seit Aufhebung der Covid-19-Pandemiebeschränkungen (<u>seit 07.04.2023</u>) schätzungsweise durchgeführt? Bitte antworten Sie frei:

Anzahl der Videosprechstunden insgesamt: ____

IV.9. Wie viele verschiedenen Patient*innen haben Sie insgesamt seit Aufhebung der Covid-19-Pandemiebeschränkungen (<u>seit 07.04.2023</u>) schätzungsweise per Videosprechstunde behandelt? Bitte antworten Sie frei:

Anzahl Patient*innen mit mind. 1 Videosprechstunde: ____

IV.10. Wie viele Videosprechstunden fanden schätzungsweise im Durchschnitt bei Ihnen wöchentlich seit Aufhebung der Covid-19-Pandemiebeschränkungen (<u>seit 07.04.2023</u>) statt?

Bitte antworten Sie frei: ____

IV.11. Wie hoch schätzen Sie den prozentualen Anteil der Videosprechstunde seit Aufhebung der Covid-19-Pandemiebeschränkungen (<u>seit 07.04.2023</u>) an Ihrem wöchentlichen Stundenkontingent ein?

Freie Schätzung in Prozent: ____

IV.12. Wie viele Ihrer aktuellen Patient*innen behandeln Sie <u>derzeit</u> per Videosprechstunde?

Aktuelle Anzahl der Patient*innen in Ihrer Praxis insgesamt: ____
davon Anzahl Patient*innen mit mind. 1 Videosprechstunde: ____

IV.13. In welchem Rahmen wenden Sie die Videosprechstunde seit Aufhebung der Covid-19-Pandemiebeschränkungen (seit 07.04.2023) an?

- ☐ derzeit in keinem Rahmen
- ☐ Akutbehandlung
- ☐ Kurzzeittherapie
- ☐ Langzeittherapie
- ☐ Rezidivprophylaxe
- ☐ anderer Rahmen, nämlich: ____

IV.14. Welche Störungsbilder haben Sie bisher im Rahmen der Videosprechstunde behandelt? *(Mehrfachantworten möglich)*

- ☐ Angststörungen
- ☐ Zwangsstörungen
- ☐ Depression
- ☐ Manien
- ☐ bipolare affektive Störungen
- ☐ Essstörungen
- ☐ PTBS
- ☐ Anpassungsstörungen
- ☐ Somatoforme Störungen
- ☐ Suchtstörungen
- ☐ Schizophrenien oder wahnhafte Störungen
- ☐ andere Störungen, nämlich: ____
  ____

IV.15. Wie würden Sie Ihre Erfahrungen mit der Videosprechstunde am ehesten beschreiben? *(Mehrfachantworten möglich)*

- ☐ Die Videosprechstunde konnte zum weiteren positiven Behandlungsverlauf beitragen.
- ☐ Die Videosprechstunde wurde von den Patient*innen vorrangig positiv wahrgenommen.
- ☐ Es ergaben sich positive Effekte, welche vorab nicht absehbar waren.
- ☐ Es waren weder positive noch negative Effekte für Sie erkennbar.
- ☐ Die Videosprechstunde wirkte sich negativ auf den Behandlungsverlauf aus.
- ☐ Sie selbst fühlten sich überfordert mit dem Ablauf und waren unzufrieden.
- ☐ Sie erhielten überwiegend negative Rückmeldungen von Ihren Patient*innen.
- ☐ eine andere, nämlich: ____
  ____

IV.16. Welche Erschwernisse erleben Sie bei der Durchführung der Videosprechstunde? *(Mehrfachantworten möglich)*

☐ schlechte Internetverbindung und häufige Störungen

☐ eigene schlechte technische Ausstattung (Webcam, Mikrophon etc.)

☐ patientenseitige schlechte technische Ausstattung (Webcam, Mikrophon etc.)

☐ Schwierigkeiten einen passenden zertifizierten Anbieter zu finden

☐ Beziehungsarbeit mit Patient*innen gestaltet sich schwierig

☐ fehlender Blickkontakt führt zu Verunsicherung

☐ anstrengender als Präsenztherapie

☐ Beeinträchtigung der nonverbalen Kommunikation (Mimik, Gestik)

☐ Beeinträchtigung der Wahrnehmung (Bildqualität, Stimmmelodie)

☐ Behandlungsplanung ist schwierig

☐ fehlende Spontanität

☐ Einschränkung von therapeutischen Techniken

☐ Risiko, dass Vermeidungsverhalten (z.B. Vermeidung von öffentlichen Verkehrsmitteln, Rückzugsverhalten) verstärkt wird

☐ sonstige: ____
____

IV.17. Haben Sie schon einmal eine*n Patient*in ausschließlich mit der Videosprechstunde behandelt (abgesehen von Sprechstunden und Probatorik?

☐ ja

☐ nein (immer in Kombination mit Präsenzsitzungen)

IV.18. Werden Sie auch in Zukunft weiter mit der Videosprechstunde arbeiten wollen?

☐ ja

☐ nein

☐ noch unsicher

*Die folgenden Fragen dürfen wieder von **allen Studienteilnehmer*innen** beantwortet werden!*

IV.19. Wie würden Sie die Wirksamkeit der Videosprechstunde im Vergleich zur Präsenz-Therapiesitzung einschätzen?

- ☐ viel besser
- ☐ besser
- ☐ gleich gut
- ☐ schlechter
- ☐ viel schlechter

IV.20. Für welche Störungsbilder würden Sie die Videosprechstunde prinzipiell als sinnvoll erachten?

- ☐ für alle Störungsbilder *ungeeignet*
- ☐ für alle Störungsbilder *geeignet*
- ☐ nur für folgende Störungsbilder geeignet: *(Mehrfachantworten möglich)*
  - o Angststörungen
  - o Zwangsstörungen
  - o Depressionen
  - o Manien
  - o Bipolare affektive Störung
  - o Essstörungen
  - o Persönlichkeitsstörungen
  - o PTBS
  - o Anpassungsstörungen
  - o Somatoforme Störungen
  - o Suchtstörungen
  - o Schizophrenien oder wahnhafte Störungen
  - o Andere Störungen: ____

IV.21. Für welche Störungsbilder würden Sie keine Videosprechstunde anbieten? *(Mehrfachantworten möglich)*

☐ für alle Störungsbilder *ungeeignet*

für folgende Störungsbilder ungeeignet: (Mehrfachantworten möglich)

☐ Angststörungen
☐ Zwangsstörungen
☐ Depressionen
☐ Manien
☐ Bipolare affektive Störung
☐ Essstörungen
☐ Persönlichkeitsstörungen
☐ PTBS
☐ Anpassungsstörungen
☐ Somatoforme Störungen
☐ Suchtstörungen
☐ Schizophrenien oder wahnhafte Störungen
☐ Andere Störungen: ____

IV.22. Welche organisatorische, technische oder fachliche Verbesserung der Videosprechstunde würden Sie sich für die Zukunft wünschen? Antworten Sie bitte frei!

IV.23. Welche Regelung der Kassenärztlichen Bundesvereinigung (KBV) empfinden Sie in Bezug auf die Videosprechstunde als überflüssig? *(Mehrfachantworten möglich)*

☐ Psychotherapeutische Sprechstunden dürfen nicht per Videosprechstunde stattfinden
☐ Probatorische Sitzungen dürfen nicht per Videosprechstunde stattfinden
☐ Obergrenze von 30 Prozent für alle per Video möglichen Leistungen nach der Psychotherapie-Richtlinie, die in einem Quartal abgerechnet werden
☐ Obergrenze von 30 Prozent aller Akutbehandlungen per Video
☐ Sonstiges: ____
____
____

IV.24. Hat sich Ihre persönliche Einstellung gegenüber dem Einsatz der Videosprechstunde nach Aufhebung der Covid-19-Pandemiebeschränkungen (seit 07.04.2023) verändert?

☐ Ich bin bei meiner Einstellung geblieben, dass die Videosprechstunde auch weiterhin ein Bestandteil meiner therapeutischen Arbeit sein wird.

☐ Ich bin bei meiner Einstellung geblieben, dass die Videosprechstunde auch weiterhin kein Bestandteil meiner therapeutischen Arbeit sein wird.

☐ Ich vertrete jetzt eine andere Einstellung hinsichtlich der Nutzung der Videosprechstunden und werde diese verstärkt nutzen.

☐ Ich vertrete jetzt eine andere Einstellung hinsichtlich der Nutzung der Videosprechstunden und werde diese zukünftig nicht mehr nutzen.

☐ Ich bin mir nach wie vor noch nicht sicher, ob ich in Zukunft mit der Videosprechstunde arbeiten werde.

IV.25. In welchem Rahmen halten Sie die Durchführung einer Videosprechstunde generell für sinnvoll? *(Mehrfachantworten möglich)*

☐ in keinem Rahmen

☐ Sprechstunde

☐ Probatorik

☐ Akutbehandlung

☐ vollständige Therapie (anstatt Präsenzpsychotherapie)

☐ begleitend zur Präsenztherapie („gemischte Therapie“)

☐ Rezidivprophylaxe

☐ Gruppentherapie

☐ Aufnahme/Überleitung vom stationären zum ambulanten Setting

☐ in einem weiteren Rahmen, nämlich: ________________________________

IV.26. Welche weiteren digitalen Angebote nutzen Sie im Rahmen Ihrer Therapien? *(Mehrfachantworten möglich)*

☐ keine

☐ Apps (z.B. deprexis)

☐ Virtual Reality

☐ sonstige: ____

IV.27. Welche Form von Unterstützung würden Sie sich bezüglich der Videosprechstunde wünschen? *(Mehrfachantworten möglich)*

☐ technische

☐ politische

☐ Fortbildungen

☐ Supervision

☐ Intervision/ Peer-Gruppen

☐ keine

☐ sonstige: ____

*Folgende Fragen beziehen sich auf die Nutzung der Videosprechstunde im Rahmen der **Gruppentherapie**.*

IV.28. Besitzen Sie die Zusatzqualifikation „Gruppentherapie"?

- ☐ ja
- ☐ nein

*Wenn die letzte Frage (IV.28.) mit **JA** beantwortet haben, machen Sie bitte gleich mit **Frage Nr. IV.29.** weiter!*

*Wenn Sie die letzte Frage (IV.28.) mit **NEIN** beantwortet haben, machen Sie bitte mit **Frage Nr. V.1.** auf Seite 17 weiter!*

IV.29. Wie viele Gruppentherapieeinheiten á 100 Minuten in Präsenz finden schätzungsweise im Durchschnitt derzeitig bei Ihnen wöchentlich statt?

Bitte antworten Sie frei: ____

IV.30. Wie viele Gruppentherapieeinheiten á 100 Minuten per Videosprechstunde finden schätzungsweise im Durchschnitt derzeitig bei Ihnen wöchentlich statt?

Bitte antworten Sie frei: ____

IV.31. Wie viele Gruppentherapieeinheiten á 100 Minuten haben Sie bereits per Videosprechstunde durchgeführt?

freie Schätzung Anzahl Gruppentherapieeinheiten á 100 Min.: ____

IV.32. Wie waren Ihre Erfahrungen mit der Videosprechstunde im Rahmen der Gruppentherapie bisher?

- ☐ sehr positiv
- ☐ positiv
- ☐ gemischt
- ☐ negativ
- ☐ sehr negativ

IV.33. Für welche Versorgungsangebote nutzen Sie die Gruppentherapie per Videosprechstunde (z.B. Gruppentherapeutische Grundversorgung, Gruppentherapie, Kombinationsbehandlung)? Bitte antworten Sie frei!

V. Ergänzende Fragen

*Bitte versuchen Sie, die folgenden Fragen zu beantworten, **auch wenn Sie bisher keine Videosprechstunde durchgeführt haben und/oder keinen Einsatz der Videosprechstunde in Zukunft planen**!*
*Folgende Fragen beziehen sich auf die Nutzung der Videosprechstunde im **Einzelsetting.***

V.1. Ich habe schon eine Fortbildung zur Videosprechstunde besucht.

☐ ja
☐ nein

V.2. Ich sehe auf meiner Seite Fortbildungsbedarf zur Videosprechstunde.

☐ stimmt sehr
☐ stimmt etwas
☐ stimmt nicht ganz
☐ stimmt überhaupt nicht

V.3. Die Regularien zum Einsatz der Videosprechstunde sind mir bekannt.

☐ stimmt sehr
☐ stimmt etwas
☐ stimmt nicht ganz
☐ stimmt überhaupt nicht

V.4. Die Regularien zum Einsatz der Videosprechstunde finde ich hilfreich.

☐ stimmt sehr
☐ stimmt etwas
☐ stimmt nicht ganz
☐ stimmt überhaupt nicht

V.5. Die Regularien zum Einsatz der Videosprechstunde schränken mich ein.

☐ stimmt sehr
☐ stimmt etwas
☐ stimmt nicht ganz
☐ stimmt überhaupt nicht

V.6. Die Videosprechstunde hat einen persönlichen Nutzen für mich als Psychotherapeut*in.

☐ stimmt sehr
☐ stimmt etwas
☐ stimmt nicht ganz
☐ stimmt überhaupt nicht

V.7. Wie sieht dieser persönliche Nutzen für Sie aus? Bitte antworten Sie frei!

V.8. Die Videosprechstunde nützt den Patient*innen in manchen Punkten.

- ☐ stimmt sehr
- ☐ stimmt etwas
- ☐ stimmt nicht ganz
- ☐ stimmt überhaupt nicht

V.9. Wie sieht dieser persönliche Nutzen für die Patient*innen aus? Bitte antworten Sie frei!

V.10. Meine Patient*innen lehnen die Videosprechstunde ab.

- ☐ stimmt sehr
- ☐ stimmt etwas
- ☐ stimmt nicht ganz
- ☐ stimmt überhaupt nicht

V.11. Ich finde, dass die Videosprechstunde für Patient*innen gut funktioniert.

- ☐ stimme überhaupt nicht zu
- ☐ stimme nicht zu
- ☐ neutral
- ☐ stimme zu
- ☐ stimme völlig zu

V.12. Die Qualität der Therapie per Videosprechstunde ist genauso gut wie bei persönlicher Therapie vor Ort.

- ☐ stimme überhaupt nicht zu
- ☐ stimme nicht zu
- ☐ neutral
- ☐ stimme zu
- ☐ stimme völlig zu

V.13. Durch die Nutzung der Videosprechstunde spare ich Zeit und/oder Geld.

- ☐ stimme überhaupt nicht zu
- ☐ stimme nicht zu
- ☐ neutral
- ☐ stimme zu
- ☐ stimme völlig zu

V.14. Menschen, deren Meinung ich schätze, denken, dass ich die Videosprechstunde nutzen sollte.

- ☐ stimme überhaupt nicht zu
- ☐ stimme nicht zu
- ☐ neutral
- ☐ stimme zu
- ☐ stimme völlig zu

V.15. Ich mache mir Sorgen, dass es schwierig sein könnte, mich mit meinen Patient*innen per Videosprechstunde verbunden zu fühlen.

- ☐ stimme überhaupt nicht zu
- ☐ stimme nicht zu
- ☐ neutral
- ☐ stimme zu
- ☐ stimme völlig zu

V.16. Ich finde es einfach, Psychotherapie per Videosprechstunde anzubieten.

- ☐ stimme überhaupt nicht zu
- ☐ stimme nicht zu
- ☐ neutral
- ☐ stimme zu
- ☐ stimme völlig zu

V.17. Ich beabsichtige, die Videosprechstunde in Zukunft zu nutzen.

- ☐ stimme überhaupt nicht zu
- ☐ stimme nicht zu
- ☐ neutral
- ☐ stimme zu
- ☐ stimme völlig zu

V.18. Meine Landespsychotherapeutenkammer unterstützt die Videosprechstunde.

- ☐ stimme überhaupt nicht zu
- ☐ stimme nicht zu
- ☐ neutral
- ☐ stimme zu
- ☐ stimme völlig zu

V.19. Ich mache mir Sorgen, ob wir Emotionen online kommunizieren können.

- ☐ stimme überhaupt nicht zu
- ☐ stimme nicht zu
- ☐ neutral
- ☐ stimme zu
- ☐ stimme völlig zu

V.20. Ich plane, die Videosprechstunde in Zukunft zu nutzen.

- ☐ stimme überhaupt nicht zu
- ☐ stimme nicht zu
- ☐ neutral
- ☐ stimme zu
- ☐ stimme völlig zu

V.21. Ich habe Bedenken bezüglich der Nutzung der Videosprechstunde.

- ☐ stimme überhaupt nicht zu
- ☐ stimme nicht zu
- ☐ neutral
- ☐ stimme zu
- ☐ stimme völlig zu

V.22. Die Verwendung der Technologie für die Videosprechstunde ist klar und verständlich.

- ☐ stimme überhaupt nicht zu
- ☐ stimme nicht zu
- ☐ neutral
- ☐ stimme zu
- ☐ stimme völlig zu

V.23. Die Arbeit per Videosprechstunde ist bequemer.

- ☐ stimme überhaupt nicht zu
- ☐ stimme nicht zu
- ☐ neutral
- ☐ stimme zu
- ☐ stimme völlig zu

V.24. Es ist leicht zu erlernen, wie man eine Videosprechstunde anbietet.

- ☐ stimme überhaupt nicht zu
- ☐ stimme nicht zu
- ☐ neutral
- ☐ stimme zu
- ☐ stimme völlig zu

V.25. Mir macht es Freude, die Videosprechstunde durchzuführen.

- ☐ stimme überhaupt nicht zu
- ☐ stimme nicht zu
- ☐ neutral
- ☐ stimme zu
- ☐ stimme völlig zu

V.26. Menschen, die mir wichtig sind, denken, dass ich die Videosprechstunde anbieten sollte.

- ☐ stimme überhaupt nicht zu
- ☐ stimme nicht zu
- ☐ neutral
- ☐ stimme zu
- ☐ stimme völlig zu

V.27. Die Nutzung der Videosprechstunde ist eine gute Idee.

- ☐ stimme überhaupt nicht zu
- ☐ stimme nicht zu
- ☐ neutral
- ☐ stimme zu
- ☐ stimme völlig zu

V.28. Ich verfüge über die erforderlichen fachlichen und technischen Kenntnisse, um die Videosprechstunde durchführen zu können.

- ☐ stimme überhaupt nicht zu
- ☐ stimme nicht zu
- ☐ neutral
- ☐ stimme zu
- ☐ stimme völlig zu

V.29. Kolleg*innen und anerkannte Expert*innen unterstützen die Videosprechstunde.

- ☐ stimme überhaupt nicht zu
- ☐ stimme nicht zu
- ☐ neutral
- ☐ stimme zu
- ☐ stimme völlig zu

V.30. Die Videosprechstunde wirkt für mich irgendwie abschreckend.

- ☐ stimme überhaupt nicht zu
- ☐ stimme nicht zu
- ☐ neutral
- ☐ stimme zu
- ☐ stimme völlig zu

V.31. Die Videosprechstunde ist nicht kompatibel mit der Art und Weise, wie ich normalerweise eine Therapie durchführe.

- ☐ stimme überhaupt nicht zu
- ☐ stimme nicht zu
- ☐ neutral
- ☐ stimme zu
- ☐ stimme völlig zu

VI. Erfassung der tatsächlich genutzten Videosprechstunden

*Folgende Fragen sind bitte von **allen Studienteilnehmer*innen** zum Zwecke der genauen Quantifizierung der tatsächlich in Anspruch genommenen Videosprechstunden zu beantworten.*

VI.1. Seit 07.04.2023 tatsächlich durchgeführte Videosprechstunden im Einzelsetting (á 50 Min.):

- ☐ 0
- ☐ 1-4
- ☐ 5-20
- ☐ 21-40
- ☐ 41-80
- ☐ 81-160
- ☐ 160-320
- ☐ 321 und mehr

VI.2. Datum der letzten durchgeführten Videosprechstunde im Einzelsetting (á 50 Min.): ***TT MM JJ***

- ☐ trifft nicht zu

VI.3. Bitte geben Sie das heutige Datum an. ***TT MM JJ***

Vielen Dank für Ihre Mitarbeit!
