# Supplementary material for: Postpandemic Use of Video-Based Psychotherapy Among German Outpatient Psychotherapists: Repeated Cross-Sectional and Partially Longitudinal Survey Study
Source: J Med Internet Res. 2026 Jul 31;28:e82972. doi: 10.2196/82972 (PMC13426897; doi:10.2196/82972)
Supplement: Multimedia Appendix 3 [file jmir-v28-e82972-s003.docx]

### **Multimedia Appendix 3**

**Table S1:** Internal Consistency of UTAUT-T Subscales (T2 Sample, N = 292).

| **Scale** | **No. of items** | **Cronbach α** | **mean (SD)** | **Example Item** |
| --- | --- | --- | --- | --- |
| Therapy Quality Expectation | 9 | .901 | 3.07 (0.37) | The quality of VBT is the same as F2F therapy. |
| Ease of Use | 4 | .674 | 3.83 (0.19) | I find providing VBT easy. |
| Pressure from Others | 2 | .955 | 2.82 (0.04) | People who are important to me think that I should use VBT. |
| Professional Support | 2 | .489 | 3.41 (0.02) | My professional organization supports VBT. |
| Convenience | 2 | .568 | 2.57 (0.07) | Using VBT saves me time and/or money. |
| Behavioral Intention | 2 | .973 | 3.64 (0.03) | I intend to use VBT after the end of the pandemic. |

**Note.** Means represent item-level averages. Higher values indicate higher agreement with the respective construct. All items were rated on a Likert scale ranging from 1 to 5. Cronbach α reflects internal consistency at T2.

**Table S2:** Attrition Analysis (N = 371).

| **Variable** | **Test** | **Test statistic** | ***P* value** | **Effect size** |
| --- | --- | --- | --- | --- |
| Gender | *χ²* test | *χ²*(1)=0.99 | .32 | V=.05 |
| Age | *t* test | t(368)=2.95 | .003 | *d*=0.33 |
| Years working as a psychotherapist | Mann-Whitney U test | U=12450.00 | .02 | *r*=0.12 |
| Years working in outpatient practice | Mann-Whitney U test | U=13538.00 | .15 | r=0.07 |
| Cognitive behavioral therapy approach | *χ²* test | *χ²*(1)=0.02 | .90 | V=.01 |
| Psychodynamic therapy approach | *χ²* test | *χ²*(1)=0.20 | .66 | V=.02 |
| Private internet use | *χ²* test | *χ²*(4)=2.23 | .69 | V=.08 |
| Professional internet use | *χ²* test | *χ²*(4)=7.49 | .11 | V=.14 |
| Intended future use of VBT | *χ²* test | *χ²*(1)=0.41 | .52 | V=.04 |
| Perceived effectiveness (VBT vs. F2F) | *χ²* test | *χ²*(1)=0.08 | .78 | V=.02 |
| Experience with VBT | *χ²* test | *χ²*(3)=10.16 | .02 | V=.22 |
| VBT sessions  (pandemic Q2) | Mann-Whitney U test | U=4500.50 | .83 | *r*=0.02 |
| Weekly VBT sessions (pandemic Q2) | Mann-Whitney U test | U=3821.50 | .24 | *r*=0.08 |
| VBT proportion contingent  (pandemic Q2) | Mann-Whitney U test | U=4575.50 | .86 | *r*=0.01 |
| VBT sessions  (pandemic Q3) | Mann-Whitney U test | U=2515.50 | .15 | *r*=0.11 |
| Current weekly VBT sessions | Mann-Whitney U test | U=4522.00 | .68 | *r*=0.03 |
| Current VBT proportion contingent | Mann-Whitney U test | U=4456.00 | .36 | *r*=0.06 |
| Number of patients treated via VBT | Mann-Whitney U test | U=2751.00 | .74 | *r*=0.03 |

**Note.** Independent samples *t* tests were used for approximately normally distributed continuous variables (age), and Mann-Whitney U tests for non-normally distributed variables (e.g., years of professional experience, VBT use variables). Chi-square tests were used for categorical variables. Effect sizes are reported as Cohen *d* for *t* tests, r for Mann-Whitney U tests, and Cramér’s V for chi-square tests. All comparisons are based on T1 data.

**Table S3:** Between-cluster differences in additional variables (N = 292).

| **Variable** | **Test** | **Test statistic** | ***df*** | ***P* value** | **Effect size** | **Significant pairwise differences (Holm-adj. p=** |
| --- | --- | --- | --- | --- | --- | --- |
| Post-pandemic VBT usage status | *χ²* | 131.11 | 2 | <.001 | V=.67 | 1>2 (*P*=.004),  1>3 (*P*<.001),  2>3 (*P*<.001) |
| Pandemic VBT usage rate | *χ²* | 82.26 | 2 | <.001 | V=.53 | 1>3, 2>3, 1>2  (all *P*≤.006) |
| Age | Welch-ANOVA | *F*(2,154.23)=6.09 | 2 | .003 | η²=.073 | 3>1 (*P*=.002),  3>2 (*P*=.018) |
| Therapeutic orientation CBT | *χ²* | 12.42 | 2 | .002 | V=.19 | 2>3 (*P*=.001) |
| Grawe’s change mechanisms | Fisher’s exact test | - | - | <.001 | V=.27 | 1>3, 1>2, 2>3  (all *P*≤.001) |
| Awareness of VBT regulations | Kruskal-Wallis | H(2)=24.3 | 2 | <.001 | η²[H]=.08 | 1>3 (*P*<.001),  2>3 (*P*=.001) |
| Perceived regulatory restrictions | Kruskal-Wallis | H(2)=32.6 | 2 | <.001 | η²[H]=.11 | 1>3 (*P*<.001),  2>3 (*P*<.001) |
| Prior VBT training | *χ²* | 0.48 | 2 | .79 | V=0.00 | 1 vs. 2: *χ²*(1)=.041,  *P*=.839, *P*>.99;  1 vs. 3: *χ²*(1)=.464,  *P*=.496, *P*>.99;  2 vs. 3: *χ²*(1)=.291, p=.590, *P*>.99 |
| Professional internet use | Kruskal-Wallis | H(2)=52.1 | 2 | <.001 | η²[H]=.17 | 3>2 (*P*<.019),  3>1 (*P*<.001),  2>1 (*P*<.001) |
